# Curative Approach to the Treatment of Beta-Thalassemia and Sickle Cell Disease with Hematopoietic Stem Cell Transplantation

**DOI:** 10.3390/jcm15041379

**Published:** 2026-02-10

**Authors:** Ugo Testa, Germana Castelli, Elvira Pelosi

**Affiliations:** Department of Oncology, Istituto Superiore di Sanità, Viale Regina Elena 299, 00161 Rome, Italy; germana.castelli@iss.it (G.C.); elvira.pelosi56@gmail.com (E.P.)

**Keywords:** hemoglobinopathies, β-thalassemia, sickle cell disease, hematopoietic stem cell transplantation, gene therapy

## Abstract

β-thalassemia and sickle cell disease are two inherited hematological diseases due to defective hemoglobin synthesis or to the production of hemoglobin with altered properties. These two conditions have prolonged survival with modern support therapies, albeit life-long, complex, expensive and resource-consuming. Studies carried out in the last three decades have shown that allogeneic hematopoietic stem cell transplantation (allo-HSCT) and gene therapy may offer a curative approach for these diseases. Allo-HSCT should be performed early in life to reduce disease-related complications like irreversible tissue damage due to iron overload in patients with transfusion-dependent β-thalassemia (TDT) and systemic vasculopathy in patients with sickle cell disease (SCD). HSCTs from a matched-sibling donor or a matched-unrelated donor represent the best therapeutic option; however, haploidentical HSCT in both TDT and SCD is now increasingly performed as a valuable and viable option for a larger number of these patients. An alternative curative strategy is based on gene therapy. These curative approaches, particularly those of gene therapy, are available only in a part of the world. Gene therapy diffusion is strongly limited by its high technological and infrastructure requirements and its very high cost. Criteria must be defined for the optimal selection of TDT and SCD patients for allo-HSCT or gene therapy.

## 1. Introduction

Hemoglobin is the main protein responsible for oxygen transportation in the human body and the major component of red blood cells. The adult HbA (α_2_β_2_) is a tetrameric protein whose coding genes are in two separate globin gene cluster families sited on different chromosomes. During fetal life, the fetal hemoglobin (α_2_γ_2_) is the predominant hemoglobin, and at birth, HbF is progressively replaced by HbA. When compared to HbA, HbF has a higher affinity for oxygen. The P50 of HbF is lower than the P50 of HbA; this higher affinity of HbF for oxygen is important to obtain oxygen from maternal circulation. This property of HbF is important and was exploited in some studies of gene therapy.

Hemoglobinopathies, the genetic diseases caused by mutations of globin genes, represent the most common monogenic disorders worldwide. The genetic causes of hemoglobinopathies are represented by DNA mutations in or near the globin genes. These DNA variants may cause a defect in globin synthesis (thalassemia) or the synthesis of a mutant, defective hemoglobin, causing diseases such as sickle cell disease (SCD).

The most frequent hemoglobinopathies are β-thalassemia and SCD.

Beta-thalassemias are a group of recessively inherited hemoglobin disorders characterized by reduced or absent production of hemoglobin and chronic anemia. The evolutionary association between the thalassemia carrier condition and resistance to malaria explains the geographic distribution of the disease involving world areas such as the Mediterranean basin, the Middle East, Southeast Asia and sub-Saharan Africa. More than 350 mutations are known for β-thalassemia; however, only 20 β-thalassemia mutations account for more than 80% of the β-thalassemia mutations across the globe. Thalassemia affected over 1,300,000 people worldwide in 2021, and the WHO declared it a major health concern [1]. Globally, approximately 60,000 newborns are born with β-thalassemia major per year, with the majority living in developing countries [2]. β-thalassemia carriers account for approximately 3% of the global population. The disease is characterized by absent/low β-globin synthesis, with consequent large excess of free α-chains that are unstable and precipitate in developing erythroid cells, causing oxidative damage and premature cell death in bone marrow (dyserythropoiesis) [3]. This pathologic mechanism determines a condition of severe anemia, requiring continuous transfusion support [3]. Advances in therapeutic support have consistently improved the life expectancy of individuals with β-thalassemia. Allogeneic hematopoietic stem cell transplantation (HSCT) from compatible healthy donors or gene therapy are the two curative approaches for β-thalassemia.

SCD is a recessive hereditary disorder caused by a point mutation responsible for the substitution of Glu6 with Val in the beta globin gene, leading to the production of the abnormal hemoglobin (HbS) that has the tendency to polymerize, with consequent erythrocyte sickling, vaso-occlusion and multi-organ damage [4,5]. The incidence of SCD is estimated to be 515,000 neonates each year, the majority in sub-Saharan Africa, which accounts for nearly 80% of global cases [6]. In 2021, an estimated 7.74 million people were living with SCD globally [6]. SCD is characterized by considerable phenotypic complexity. At the clinical level, several acute complications, such as acute chest syndromes, stroke and acute pain events, are commonly observed; chronic complications can damage all organs. In spite of consistent progress in the supportive care of SCD patients, the prognosis remains poor, and the only curative approaches are represented by allo-HSCT or gene therapy.

Studies carried out in the last two decades have shown very significant improvement in curative approaches to β-thalassemia and SCD based on allo-HSCT and gene therapy. This review analyzes these studies and discusses the problem of therapeutic choices for β-thalassemic and SCD patients.

## 2. Materials and Methods

The aim of this review paper is to map the existing literature on HSCT as a curative approach for the treatment of SCD and β-thalassemia. Thus, this review aims to provide an overview of the evidence supporting HSCT in SCD and β-thalassemia as a curative approach and may be considered as a scoping review. In line with the aims of a scoping review, a critical appraisal of the studies included was not performed [7,8]. This literature review aligns with the criteria of the Preferred Reporting Items for Systematic Review and Meta-Analyses extension for scoping reviews (PRISMA-ScR) [9].

Articles or congress abstracts were included in the literature search if they were (i) about HSCT, gene therapy, SCD or β-thalassemia; (ii) original reports of data written in English, with full-text availability on research studies or clinical outcomes and psychosocial outcomes; and (iii) focused on SCD and/or β-thalassemia. The PubMed, EMBASE, and Health Business Elite databases were searched until December 2025 using key terms related to allo-HSCT, gene therapy, SCD and β-thalassemia.

In addition to the literature articles indexed on international databases, an important source of scientific information was derived from the abstracts of the Congress of the American Society of Hematology (ASH Meeting) and of the Congress of the European Hematology Association. Some data on allo-HSCT in β-thalassemic patients were obtained from the Guidelines for the Management of Transfusion-Dependent β-thalassemia (Internet Book), 5th edition, Nicosia, Cyprus, Thalassemia International Federation, 2025 [10].

Each document was analyzed by at least two of the three authors of the present article.

## 3. Hematopoietic Stem Cell Transplantation in SCD

The widespread adoption of HSCT for patients with SCD is limited by the scarce availability of fully matched bone marrow donors and by the transplant-related morbidity and mortality [11]. The greatest success is achieved when HSCT is made using an HLA-matched-related donor (MRD) and a myeloablative conditioning regimen in young SCD patients. Various factors limit this approach, represented not only by the scarce donor availability but also by age at HSCT, associated organ dysfunction, and resource accessibility [12]. HLA-matched HSCT using an HLA-identical sibling donor (8/8 match) is the gold standard and is associated with overall survival rates ranging from 88% to 97% and event-free survival rates ranging from 81% to 92%.

Many studies have consistently shown that HLA-matched sibling donor transplantation is a highly effective, potentially curative treatment for SCD, particularly in children and young adults.

### 3.1. HSCT with HLA-Matched Donors in SCD Patients

Finding a matched donor is a significant obstacle, as fewer than 15–20% of patients have an HLA-identical sibling donor. In a recent study on the evaluation of 128 SCD patients undergoing allo-HSCT, a matched HLA sibling donor was observed in about 50% of cases; in this study, the number of siblings per patient was 2 [13]. Allo-HSCT requires adequate conditioning to ablate recipient HSCs in the bone marrow and to replace these cells by transplantation of donor WT-HSCs that must engraft and repopulate bone marrow, and an immunosuppression sufficient to prevent graft rejection. Allo-HSCT with a myeloablative regimen offers the advantage of being associated with a higher frequency of full-donor chimerism compared to allo-HSCT with non-myeloablative conditioning. Several studies have confirmed the curative effect of allo-HSCT with matched-related donors after a myeloablative conditioning regimen (Table 1) In SCD patients with severe disease and with an age comprised between 2 and 20 years, MRD myeloablative allo-HSCT resulted in a high rate of EFS and OS (>95%) with a low GVHD and a low rate of transplantation-related mortality (due to GVHD) [14] (Table 1). Thus, HLA-identical sibling HSCT after myeloablative conditioning with anti-thymocyte globulin should be considered as a standard of care for SCD children with severe disease, primarily in those with life-threatening complications like stroke or at high-risk of neurological complications, recurrent chest syndrome, frequent pain crises despite therapy, severe organ damage, or needing consistent transfusion support [14].

In a study of 736 SCD patients who underwent HSCT from an HLA-matched sibling donor between 1986 and 2017, OS and EFS were better in younger patients [12]. Particularly, SCD patients have been subdivided into the age groups: 0–5 years; 6–15 years; and >15 years [15]. The 4-year OS and EFS rates in these three age groups were: 100%, 95% and 88%, respectively, for OS, and 93%, 89% and 81%, respectively, for EFS [15]. Furthermore, in patients with age > 15 years, aGVHD and cGVHD incidence increased up to 17% and 20%, respectively [15]. These findings support an early referral of SCD patients to HSCT [15].

A large retrospective international survey based on the analysis of 872 SCD patients (760 children and 113 adults) who have undergone MRD allo-HSCT after myeloablative conditioning showed a 5-year EFS and OS of 91.4% and 92.9%, respectively [16]. EFS was lower with increasing age, the rate of graft failure was low (2.3%) and the death rate was 7% (mostly related to infectious events) [16]. It is of interest to note that, despite the excellent 5-year EFS and OS, in 10% of patients who died, death occurred beyond 5 years, an event seemingly related to both end-organ damage from SCD and transplant [16]. Bernaudin and coworkers reported the long-term outcomes of 234 SCD patients under 30 years of age who had been transplanted after myeloablative conditioning; all patients received matched-sibling donor stem cell transplantation [17]. The 5-year EFS was 97.9% [13]. At day 100, acute GVHD was observed in 20.1% of cases; chronic GVHD was observed in 24 patients, but was extensive in only six cases [17]. At 1 year, 44% of patients displayed mixed chimerism; no SCD-related events occurred in these patients with mixed chimerism, even those with 15–20% donor cells [17]. The major long-term concern with SCD patients undergoing myeloablative allo-HSCT was infertility risk in both genders [17].

Walters et al. compared outcomes prospectively through 2 years after biologic assignment to a donor or no donor (standard of care) arm based on the availability of a donor, either an HLA-matched sibling or an unrelated donor [18]. The study compared the survival of these two groups of patients 2 years after biologic assignment [18]. The study enrolled a total of 113 participants, 28 in the donor arm and 85 in the no-donor arm; the 2-year probabilities of 2-year survival were 89% and 93%, in the donor vs. no-donor arms [18]. Assignment to the donor arm led to improvement in vascular occlusive events, fatigue and social function [18]. These observations showed that short-term mortality after HSCT was not excessive when compared with standard of care for SCD; in the long term, it is expected that there will be an advantage in survival, as well as in quality of life in the donor arm compared to the no-donor arm [18].

These studies have now contributed to establishing that age at transplantation is a key determinant of the outcomes of allo-HSCT in SCD patients. In fact, younger SCD patients typically have fewer SCD-related complications (like organ damage) before HSCT, making them better candidates for intensive conditioning. Older SCD patients are more susceptible to toxicities from myeloablative conditioning regimens, leading to modifications of conditioning regimens, such as reduced-intensity or non-myeloablative protocols.

### 3.2. HSCT in SCD Patients Using Non-Myeloablative Conditioning Regimens

Conditioning regimens are subdivided into three categories: (a) myeloablative conditioning (MAC), (b) reduced-intensity conditioning (RIC), and (c) non-myeloablative conditioning (NMA). These three different categories are distinguished by the duration of cytopenia and the requirement for stem cell (SC) support: MAC regimens cause irreversible cytopenia and require HSC support. NMA regimens cause minimal cytopenia and can also be given without HSC support. RIC regimens have intermediate criteria between MAC and NMA regimens: they cause cytopenia of variable duration and require stem cell support, although cytopenia may not be irreversible [19].

It was shown that a minimum state of peripheral blood chimerism of 20% is sufficient to reverse the sickle phenotype [20]. The relationship between chimerism, HbS levels, and symptomatic disease was retrospectively correlated in 95 SCD patients who had chimerism reports available at 1 and 2 years after transplantation [20]. Among the patients with mixed chimerism, the lowest documented chimerism without symptomatic disease was 26% [17]. These observations suggest that stable donor chimerism greater than 25% is associated with resolution of SCD-related symptoms [21].

In line with these observations, non-myeloablative (NMA) conditioning was developed to extend transplantation opportunities to SCD patients unable to tolerate the toxicities induced by myeloablative regimens, inducing a condition of stable mixed donor chimerism and reversing the pathologic effect induced by SCD [22,23,24].

Achieving long-term donor engraftment and minimizing transplantation-related complications in NMA allo-HSCT requires an adequate regimen of immunosuppression. In this context, the National Heart, Lung, and Blood Institute (NHLBI) NMA regimen with Alemtuzumab, low-dose radiation, and Sirolimus in individuals with severe SCD undergoing MRD allo-HSCT showed a high and sustained engraftment, favorable overall and disease-free survival, low regimen-related toxicity, no-transplantation-related mortality, no acute or chronic GVHD, and secondary graft failure in 13% of cases [22,24]. In this regimen, SCD patients received Alemtuzumab at increasing doses at day −7 to −3 before infusion of allo-PBSCs from G-CSF-mobilized HLA-identical sibling donor, total body irradiation of 300 rad two days before transplantation, and Sirolimus administration starting from day 1 before transplantation to two years after transplantation [22,23,24]. Inaman and coworkers have reported the outcomes observed in 91 adult SCD patients with severe disease who were transplanted with MRDs after the NHLBI NMA regimen: some patients received the standard conditioning regimen, and other patients received the same conditioning regimen, preceded by a preconditioning immunodepletion [25]. At a median follow-up of 7.3 years, OS was 90%, SCD-free survival was 85%, and mixed myeloid chimerism was 43%; outcomes were comparable in the two conditioning regimen protocols [25]. The graft failure rate observed in this protocol can be reduced by adding a preconditioning immunodepletion based on Pentostatin and oral Cyclophosphamide administration [25]. Hematologic malignancy or abnormal cytogenetics developed in 7 out of 91 patients [25].

For adult SCD patients, myeloablative regimens are not feasible due to existing organ damage [12,26]. Thus, several reduced-intensity non-myeloablative regimens were used, combining myelosuppressive agents (such as Busulfan, Threosulfan and Thiotepa) and immunosuppressive agents (such as Cyclophosphamide and Fludarabine) with or without low-dose total body irradiation (TBI) [12,26].

Alzahrani et al. reported the results of a clinical study carried out in 122 SCD patients, predominantly adult patients (median age 29 years, with only 9% of patients with <18 years), undergoing MRD allo-HSCT with G-CSF mobilized PB HSCs, after non-myeloablative conditioning based on TBI (300 cGy) and alemtuzumab [27]. Overall survival at 1 year and at 5 years was 98% and 93%, respectively [27]. A total of 16 patients (13%) experienced graft failure: 13 primary and 3 secondary failures [27]. Median neutrophil and platelet engraftment occurred on days 22 and 19, respectively [27]. Mean donor myeloid and lymphoid (CD3) chimerisms at 5-year post-transplant were 88% and 53%, respectively [27]. Two patients developed acute-GVHD of grades 1 and 2, with no chronic GVHD [27]. No patient developed solid cancer; two patients developed a myelodysplastic syndrome, and one patient developed chronic myeloid leukemia [27]. Hematological parameters markedly improved post-HSCT; SCD-related organ damage stopped progressing [27]. Interestingly, G-CSF administration post-HSCT to adult SCD patients may improve myeloid chimerism and may mitigate the risk of secondary graft loss [28].

More recently, Damlaj et al. reported the results observed in 200 SCD patients with severe SCD, mean age of 26 years, undergoing NMA, MRD allo-HSCT, treated between 2015 and 2021 at the Division of Hematology of Rivadh, Saudi Arabia [29]. Some of the patients received G-CSF post-transplant to accelerate neutrophil recovery. A total of 17 patients (8.5%) experienced graft failure: 3 as a primary and 14 as a secondary event [29]. A total of 76% of patients discontinued Sirolimus at the latest follow-up. The 3-year EFS and OS were 88% and 95%, respectively [29]. Multivariate analysis results showed that non-ABO incompatibility, as evidenced by the presence of alloantibodies against donor non-ABO antigens, was associated with increased risk of graft failure [30]. Patients with graft failure had inferior survival and then strategies to reduce this event are required in this setting of allo-HSCT [30]. The same authors recently reported the study of 115 SCD patients undergoing an alternative reduced-intensity conditioning regimen based on an initial 4-week administration of prednisone at alternate days (to attenuate inflammation, to promote endothelial stabilization, and to mitigate intravascular hemolysis) and then on the administration of intravenous busulfan, fludarabine, and ATG [31]. GVHD prophylaxis utilized either Cyclosporine A/Methotrexate or Tacrolimus/Methotrexate. At 1 year, the OS was 96.5%; the incidence of acute GVHD and chronic GVHD was 19% and 10%, respectively [31].

**Figure 1:**
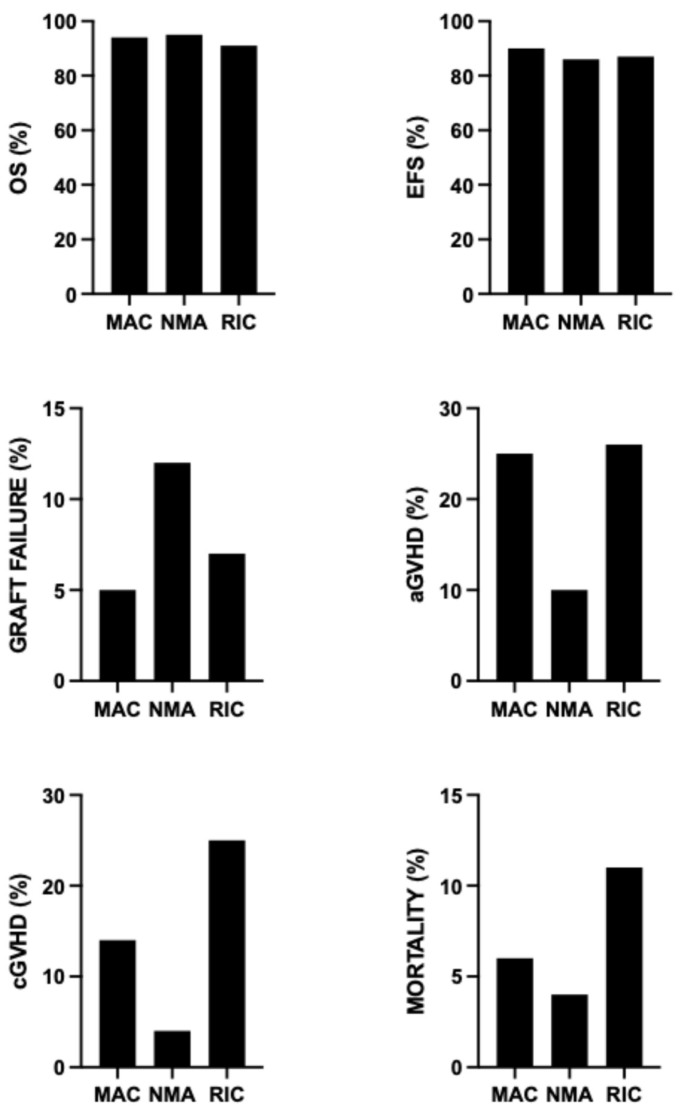
Outcomes of allo-HSCT in SCD patients who received transplantation using different conditioning regimens (MAC, NMA and RIC). Data reported in Folarin et al. 2025 [32].

A systematic review and meta-analysis of the studies of allo-HSCT performed until March 2025 analyzed the outcomes according to the different conditioning regimens classified according to their intensity in MAC, RIC and NMA, showing that: RIC was associated with a lower OS compared to MAC and NMA and with a comparable OS in MAC and NMA; EFS was lower in NMA and RIC compared to MAC; graft failure rate was higher in NMA and RIC than in MAC; aGVHD was lower in NMA and RIC than in MAC; cGVHD was higher in RIC and MAC than in NMA; and transplantation-related mortality was higher in RIC than in MAC and NMA and higher in MAC than in NMA [32] (Figure 1).

The conclusion emerging from these studies is that MAC offers high cure rates, particularly in children, but with significant toxicity, while NMA uses a less intense conditioning regimen, which reduces side effects like GVHD, making it better for adults, but increases the risk of graft failures. Thus, MAC is curative but toxic for adults; NMA is safer for adults but carries risks such as graft failure (Table 2).

### 3.3. Conditioning Regimens for Pediatric SCD Patients

A MAC regimen is the gold standard for HLA-identical sibling HSCTs despite the risk of long-term toxicity, particularly pronounced in patients who have tissue damage. In order to reduce these toxicity risks, the aim of a tailored conditioning regimen in children is to preserve fertility, while, in adults, it is to reduce toxicity in compromised patients due to underlying disease. In recent years, there was a considerable increase in the number of allo-HSCT performed both in children and adults using Treosulfan instead of Busulfan in the conditioning regimens used for SCD patients [33]. In recent years, Fuladarabine, Treosulfan and Thiotepa (FTT) emerged as a frequently adopted MAC conditioning in SCD in children and adults, taking advantage of its good tolerability to a limited endothelial toxicity and reduced blood–brain barrier passage and higher tendency for a preserved fertility [33].

The Treosulfan-based regimens are increasingly used in pediatric patients due to their more favorable toxicity profile, also including a higher likelihood of preserved fertility compared to Busulfan-based regimens. While still gonadotoxic, several studies indicate that Treosulfan is less toxic for ovarian and testicular function, particularly in prepubertal patients [34,35,36]. Particularly, these studies have shown the following: in pediatric HSCT survivors for non-malignant diseases, including SCD and β-thalassemia, Treosulfan conditioning elicited significantly higher rates of spontaneous puberty (particularly in girls) and a lower frequency of permanent gonadal failure compared to Busulfan; Busulfan-based conditioning usually leads to premature ovarian insufficiency, while Treosulfan-based regimens have shown less pronounced inhibitory effects on ovarian function; Treosulfan induced less damage to spermatogenesis compared to Busulfan; and prepubertal girls treated with Treosulfan had a high likehood of reaching spontaneous menarche than those treated with Busulfan [34,35,36].

Despite being less toxic, Treosulfan is not completely devoid of genotoxic effects. In fact, van der Stoep and coworkers showed that 28% of patients, including both males and females, experienced some form of gonadal dysfunction after Treosulfan, in many instances only transiently [35].

### 3.4. Haploidentical HSCT in SCD Patients

When an HLA-identical sibling is not available, a donor from a stem cell registry, either identifying a matched-unrelated donor or a haploidentical-related donor (frequently a parent), can be used. The main studies involving HLA-haploidentical HSCT in SCD patients are reported in Table 3. Initial studies in SCD patients provided evidence that haploidentical HSCT was an unacceptable strategy for these patients due to high rates of graft failure and death [37].

In this context, emblematic was the study carried out by Shenoy and coworkers [34]. These authors have evaluated in the context of a phase II clinical study (BMT CTN 0601) a group of 30 children with severe SCD undergoing HLA-unrelated (URD) bone marrow transplantation: a regimen of Alemtuzumab, Fludarabine and Melphalan was used using Alemtuzumab early to provide recipient immune suppression to overcome the consistent risk of graft rejection, while also mitigating toxicities associated with myeloablative agents [34]. GVHD prophylaxis included calcineurin inhibitor, short-course Methotrexate, and Methylprednisolone [38]. The 1- and 2-year EFS and OS were 76% and 69%, respectively, and 86% and 79%, respectively [27]. The day-100 incidence rate of acute GVHD II-IV was 20%, and the 1-year incidence rate of chronic GVHD was 62% (in 38% of cases, chronic GVHD was extensive) [38]. There were seven GVHD-related deaths [38]. The 5- and 8-year probabilities of OS were 68% and of EFS 61% and 57%, respectively [39]. These observations indicate that engraftment and cure were achievable with a reduced-intensity regimen following unrelated donor HSCT; however, the GVHD prophylaxis regimen was inadequate, particularly in patients over 13 years of age, compromising outcomes and increasing mortality [39].

The introduction of techniques of T-cell depletion and of treatment preventing GVHD development, such as post-transplant cyclophosphamide, improved the outcomes of haplo-HSCT in SCD patients [37]. A notable example of the significant effect of post-transplantation Cyclophosphamide in preventing GVHD is given by the study of Fitz hough et al. in patients with SCD and severe organ damage who undergo haploidentical HSCT [40]. These authors evaluated, in the context of a phase I–II trial, a non-myeloablative HLA-haploidentical PBSCT approach based on Alemtuzumab, 400 cGy total body irradiation, and increasing doses of post-transplantation Cyclophosphamide: 0 mg/kg (cohort 1), 50 mg/kg (cohort 2), and 100 mg/kg (cohort 3) [40]. No transplant-related mortality was observed, and 90% of patients remained disease-free in cohort 3 compared to 0% in cohort 1 [40]. There were no grades II to IV aGVHD or extensive cGVHD [40]. Furthermore, de la Fuente and coworkers explored haplo-HSCT in a group of 15 SCD patients with an age of inclusion from 7 to 40 years who received a conditioning regimen based on Fludarabine+Cyclophopshamide+Thiotepa+ATG+TBI and a GVHD prophylaxis based on post-transplantation Cyclophosphamide+Sirolimus+MMF [41]. At a median follow-up of 13 months, 95% of patients achieved stable engraftment with an OS of 100%; two patients had grades III-IV aGVHD, and one patient had mild cGVHD [41].

Three different strategies of haplo-HSCT have been explored in SCD patients: (i) a non-myeloablative, T-cell replete, bone marrow transplant with thiotepa and post-transplant cyclophosphamide, with the aim of achieving complete donor chimerism; (ii) a non-myeloablative, in vivo T-cell depletion, using peripheral blood stem cells with the aim of achieving a stable mixed donor–recipient chimerism; and (iii) a myeloablative, ex vivo T-cell depletion using PBSCs and advanced technology graft manipulation, with the aim of achieving complete donor chimerism [37].

In this context, two recent studies were particularly relevant. Kassim et al. reported the results of a prospective phase II study of haploidentical HSCT in SCD patients; 70 children and young SCD patients without an HLA-matched sibling donor or an available first- or second-degree haploidentical relative were enrolled; patients with a history of stroke or end-organ damage were included [41]. Conditioning was achieved with a regimen with multiple agents, including anti-thymocyte globulin, fludarabine, thiotepa, cyclophosphamide and TBI (200 centigray); GVHD prophylaxis was achieved with post-transplant cyclophosphamide [42]. At two years, EFS was 82.6%, and OS was 94.1%; all five deaths observed in the study were related to infectious complications [42]. Graft failure was observed in 11.4% of patients, who received a second haploidentical transplant; GVHD was observed in 10% of patients [42].

A second study (in the context of the phase II BMT-CTN1507 clinical trial) involving 54 adult SCD patients undergoing haploidentical bone marrow transplantation after a conditioning similar to that reported in the other study showed a 2-year EFS of 88% and an OS of 95%; two patients had graft failure (one primary and one secondary graft failure); the rate of grade III acute GVHD was 4.8% and of chronic GVHD was 22.4% (with 7% of patients with moderate/severe chronic GVHD) [43]. The median time to neutrophil and platelet recovery was 25.5 days and 34.5 days, respectively; 95% of patients achieved a condition of full donor chimerism; all patients with full donor engraftment displayed a significant improvement of hematological parameters [43].

A third study, always in the context of the BMT-CTN 1507 trial, evaluated 39 pediatric SCD patients (median age 12.5 years); the conditioning regimen was similar to that reported in the study on adult patients [44]. The results obtained in this study showed: a 2-year EFS and OS post-transplantation of 79% and 94.5%, respectively; that graft failure occurred in 15.4% of patients; that the median time to neutrophil and platelet engraftment was 22 and 31 days, respectively; that cGVHD was observed in 30% of patients; and that two deaths occurred for infectious complications [44].

Another recent study confirmed the good outcomes of haplo-HSCT in adult SCD patients [45]. In this study, 22 adult SCD patients underwent haplo-HSCT after a conditioning regimen based on anti-thymocyte globulin, fludarabine, and cyclophosphamide to prevent GVHD [45]. Engraftment was observed in 20/22 patients with full donor chimerism, while 2 patients had secondary graft failure; acute GVHD and chronic GVHD were observed in 2 and 3 patients, respectively; organ damage remained stable or improved, and no vaso-occlusive events were observed post-transplant [45].

Other studies of haplo-HSCT in SCD patients were based on the use of donor cells depleted of T-lymphocytes either by a selective T-cell depletion or by purification of the CD34^+^ cell population, enriched in HSCs and deprived in T-cells.

Foell and coworkers performed a phase II clinical trial to evaluate α/β T-cell-depleted HSCT in children and adults with SCD patients lacking sibling HLA-identical donors who have failed prior hydroxyurea therapy [46]. These investigators used a myeloablative conditioning regimen based on Fludarabine+Thiotepa+Treosulfan+ATG and a GVHD prophylaxis based on Tacrolimus and MMF [46]. The patients received CD3^+^/CD19^+^ or TCRαβ^+^/CD19^+^ PBSC grafts. After a median follow-up of 22 months, OS and EFS were both 88%, transplantation-related mortality was 12%, grades I-II and grades III-IV aGVHD were 28% and 0%, respectively, moderate or severe cGVHD were observed in 16% and 0% of cases, respectively [46]. An updated analysis of this study, extended to 29 patients, confirmed these findings [47].

Gibson reported the outcomes observed in 11 SCD patients who had received unrelated peripheral blood donor stem cell transplantation with partial T-cell depletion [44]. Conditioning included Hydroxyurea, ATG, Fludarabine, Thiotepa, and Busulfan; graft manipulation included CD3^+^/CD19^+^ depletion with 1 × 10^5^ cells/kg addback or TCRα/β^+^/CD19^+^ depletion [48]. In addition, 1-year and 3-year OS was 93.8% and 81.3%, respectively [48]. No patient developed grades III-IV aGVHD or moderate to severe cGVHD [48]. All patients receiving 10/10 HLA-compatible donors had stable engraftment, while 60% of patients receiving grafts from 9/10 HLA-compatible donors had graft failure [48].

**Table 1:** Matched-related donor HSCT in patients with SCD.

Study, Study Type and Sample Size (n)	Age Range (Year)	Conditioning Regimen	Donor Type	Overall Survival (%)	Event-Free Survival (%)	GVHD Prophylaxis	Acute GVHD (%)	Chronic GVHD (%)	Graft Failure (%)	Mortality (%)	Follow Up (Years)
Gluckman et al. [16] Retrospective (100)	0.3–54	MAC (87%) RIC (13%)	MRD	93	91	CSA, CSA+MTX, CSA+MMF	14.8	14.3	2	7	5
Bernaudin et al. [14] Retrospective (87)	2–27	MAC (Bu+CY±ATG)	MRD	93 (6 years)	86 (6 years)	CSA±MTX	20	11 (limited 9) 2.4 (extensive)	22 (without ATG) 3 (with ATG)	6.9 (5 years)	6
Bernaudin et al. [17] Retrospective (234)	22–28.9	MAC (BU+CY+ATG)	MRD	97%	94%	CSA+MTX	20.1	10.5	2.6	3	5
Eapen et al. [49] Retrospective (910)	1–48	MAC RIC NMA	MRD Haplo MUD MMUD	92	81	CNI, CNI+MTX, CNI+Sirolimus, CY post-HSCT, Ex vivo T-cell depl.	10.4	21	12.4	8.4	3
Alzahrani et al. [27] Clinical trial (122)	10–65	NMA (TBI 300 cGy Alemtuzumab)	MRD	98 (1 year) 93 (5 years)	NR.	Sirolimus	1.6	0	13	5.76	1
Damlaj et al. [29] Retrospective (200)	14–43	NMA	MRD	95% (3 years)	88%—year	Sirolimus	4	0.5	8.5	2	3
Nasiri et al. [31] Retrospective (115)	14–43	RIC	MRD	96.5 (1 year)	90	CSA+MTX TA+MTX	19	10	NR	3.5	1

NR: Not Reported.

**Table 2:** Main features of MAC and NMA/RIC allo-HSCT in SCD patients.

	MAC	NMA/RIC
Toxicity	Higher	Lower
Target Population	Mostly Children	Adults and Older Patients
Graft Failure	Lower	Higher
GVHD	Higher	Lower
Cure Rates	Better for Pediatric Patients	Better for Adult Patients

**Table 3:** Haploidentical HSCT in patients with SCD.

Study, Study Type and Sample Size (n)	Age Range (Year)	Conditioning Regimen	Donor Type	Overall Survival (%)	Event-Free Survival (%)	GVHD Prophylaxis	Acute GVHD (%)	Chronic GVHD (%)	Graft Failure (%)	Mortality (%)	Follow Up (Years)
Shenoy et al. [34] Phase II (30)	3–20	Flu+Mel+Alemtuzumab	Haplo	86 (1 year) 79 (2 years) 68 (5–8 years)	76 (1 year) 69 (2 years) 61 (5 years) 57 (8 years)	CNI+MTX+Prednisolone	28	62 (1 year) 38 extensive	14 (8 years)	24	1, 2, 5, 8
Fitzhugh et al. [36] (12)	20–56	Alemtuzumab+TBI	Haplo	92	50 0 (cohort 1) 90 (cohort 3)	CY post-HSCT+Sirolimus CY 0 (cohort 1) CY 50 mg (cohort 2) CY 100 mg (cohort 3)	8 grade I	8 limited		0	1
De la Fuente et al. [37] Phase II (18)	17–40	NMA: Llu+CY+Thio+ATG+TBI	Haplo	100	93	CV post-HSCT+ Sirolimus+MMF	13 grades III-IV	6 mild	5	0	1
Cairo et al. [46] Phase II (19)	3.3–20	BU+CY+Thio+Flu+ATG+ T-lymphocytes (add back)	Familial Haplo	90 (1 year) 84 (2 years)	90 (1 year) 84 (2 years)	None	6.2 grades II-IV	6.7 moderate- severe	0	15	2
Kassim et al. [38] Phase II (70)	8–31.3 Pediatric group (32) Adult group (38)	ATG+Flu+Thio+CY+TBI	Haplo	94 (2 years)	82.6 (2 years)	CY post-HSCT	10 grades III-IV	10 moderate- severe	11.4	7	2
Kassim et al. [39] Phase II (54)	15.5–43.2	ATG+Flu+Thio+CY+TBI	Haplo	95 (2 years)	88 (2 years)	CY post-HSCT+ Sirolimus+MMF	4.8 grade III	22.4 7 moderate-severe	4	8	2
Walters et al. [40] Phase II (39)	5–15	ATG+Flu+Thio+CY+TBI	Haplo	94.5 (2 years)	79 (2 years)	CY post-HSCT+ Sirolimus+MMT	15.5 grades II-IV 5.2 grades III-IV	30	15.4	5	2
Foell et al. [42] Phase II (25)	3–31	Treo+Thio+Flu+ATG	Haplo	88	88	CSA+MMF	28 grades I-II	16 moderate	0	12	22 months

Gilman and coworkers have reported the results of a phase II study in children and young adults with severe SCD using a reduced-intensity conditioning regimen with CD34^+^-selected, T-cell-depleted PBSC grafts. A total of 9/10 patients survived 49 months after transplantation with no sickle cell-related complications. Two patients had grades II-IV aGVHD, and one had cGVHD. Surviving patients had stable chimerism. Epstein–Barr virus-related post-transplant lymphoproliferative disorder (PTLD) occurred in three patients, and 1 patient died as a consequence of treatment of post-transplant lymphoproliferative disorder (PTLD) [49].

Cairo et al. explored another strategy of haplo-HSCT based on the use of the purified CD34^+^ cell population, enriched in HSCs and deprived of lymphoid cells [46]. Using CD34^+^ cells, they reported the results observed in 19 familial haploidentical HSCT in children and adolescent SCD patients lacking sibling HLA-identical donors and who have failed prior hydroxyurea therapy [50]. These investigators used a myeloablative conditioning regimen based on Fludarabine+Thiotepa+Treosulfan+TAG and a GVHD prophylaxis based on Tacrolimus and MMF [50]. The patients received CD3^+^/CD19^+^ or TCRα/β^+^/CD19^+^ PBSC grafts [50]. After a median follow-up of 22 months, OS and EFS were both 88%, transplantation-related mortality was 12%, grades I-II or II-IV aGVHD was 28% and 0%, respectively, and moderate or severe cGVHD was observed in 16% and 0% of cases, respectively [50].

Vallée and coworkers reported a different strategy based on the use of a reduced toxicity, myeloablative chemotherapy-based HSCT protocol with intensive immunosuppression tuned according to donor/recipient HLA mismatch, associated with high survival rates and low GVHD incidence in SCD patients [51]. In this study, 31 SCD patients (ages ranging from 2 to 22 years) with matched familial donors (MFD, 15), haploidentical familial donors (MMGD, 10), and matched-unrelated donors (MUD, 6) received allo-HSCT after a conditioning regimen with Alemtuzumab/ATG, Thiotepa, Fludarabine, and Treosulfan and post-transplantation Cyclophosphamide; after the first six patients, Treosulfan was replaced by targeted Busulfan [51]. After a follow-up of 26 months, all patients were alive and off immunosuppression [51]. Two MMFD patients experienced secondary graft failure, both after Treosulfan conditioning [51]. No grade III aGVHD nor moderate/severe cGVHD were observed [51].

The initial criteria of eligibility of SCD patients for haploidentical transplantation studies involved the enrollment of patients with overt stroke, subsequently enlarged to include patients with silent cerebral infarcts. More recently, it was proposed that incremental eligibility criteria, including also patients with life-threatening acute chest syndrome, pulmonary hypertension, persistent systemic hypertension, acute untreatable pain and repeated episodes of major priapism [52].

Shrestha et al. reported the development of a novel HLA-specific flow cytometry-based assay (HFA) as a tool to evaluate lymphocyte donor chimerism as a potential predictive indicator of engraftment outcomes. This assay utilizes cell surface markers (CD3, CD56 and HLA antibodies targeting a donor or patient-specific HLA for each case) for analysis of lymphocyte donor chimerism. This assay confirmed the strong donor chimerism for most of the adult SCD patients enrolled in the BMT CTN 1507 study [53]. HFA testing identified the two patients with scarce donor engraftment before they were diagnosed as patients with a secondary graft failure [53].

Eapen et al. reported the results of a retrospective study aiming to explore the impact of donor type and conditioning regimen intensity on allo-HSCT outcomes in a group of 910 SCD patients. The study reported the data of 90 US centers referring their results to the Center for International Blood and Marrow Transplant Research (CIBMTR). These patients underwent allo-HSCT from MRD, haploidentical-related (HAPLO), matched-unrelated (MUD) and mismatched-unrelated (MMUD) donors; different conditioning regimens were used in these patients, either myeloablative or reduced-intensity regimens or non-myeloablative regimens [54]. Conditioning regimens were considered myeloablative when busulfan was administered at concentrations greater than 150 mg/m^2^; regimens using lower doses of busulfan or melphalan were considered reduced intensity; TBI regimens (200–400 cGy) were considered non-myeloablative; myeloablative conditioning is the regimen with the highest intensity and non-myeloablative with the lowest intensity, whereas the reduced-intensity regimens fall in an intermediate intensity category [54]. Overall survival was lower in patients aged 13 years or older than in those younger than 13 years; in those who received myeloablative and RIC regimens rather than in those who received non-myeloablative regimens, and after transplantation of grafts from donors who were not HLA-matched siblings [54]. EFS was lower in recipients aged ≥13 years, those transplanted with grafts from HAPLO, MUD and MMUD compared to MRD, and those conditioned with reduced-intensity regimens compared to non-myeloablative regimens [49]. EFS did not differ between myeloablative and non-myeloablative regimens [49]. Mortality risks and graft failure were higher after any alternative donor transplantation (HAPLO, MUD or MMUD) compared to HLA-matched sibling transplantation (MRD) and resulted in lower EFS [54]. Donor type was associated with graft failure: compared with MRD, the proportion of patients with graft failure increased among recipients of HAPLO, MUD and MMUD transplants, and graft failure rate did not change between HAPLO and MUD patients [49]. Myeloablative and reduced-intensity regimens were associated with higher mortality and higher incidence of acute and chronic GVHD. GVHD incidence increased in recipients of transplants from HAPLO, MUD and MMUD compared to MRD donors [54]. The most relevant conclusions of this study are that EFS and OS are improved in SCD patients who received an allo-HSCT at age 12 years or lower and those with an MRD; for those without an MRD, different types of alternative donors seem to be equivalent. According to these observations, a risk score model to predict EFS after allo-HSCT in SCD patients was proposed. In this model, two risk factors were considered: age at transplantation and donor type [55]. Patients aged 12 or younger with an HLA-matched sibling donor were at the lowest risk score with a 3-year EFS of 92%; patients aged 13 or older with an HLS-matched sibling donor or aged 12 years or younger with an HLA-matched-unrelated donor were at intermediate risk score and display a 3-year EFS of 87%; and patients of any age with an HAPLO relative or HLA-mismatched-unrelated donor and patients aged 13 or older with an HLA-matched-unrelated donor were at high-risk score [55].

In non-myeloablative haploidentical HSCT with conditioning regimens involving TBI, the dose of TBI is a key determinant in engraftment rate. In fact, Bonas-Mende and coworkers showed that increasing the TBI dose from 200 to 400 cGy improved engraftment while maintaining the safety profile [56]. In fact, these authors explored a group of 17 patients with beta-hemoglobinopathies (12 SCD and 5 β-thalassemia) who received haploidentical HSCT after a conditioning regimen based on ATG, Fludarabine, Cyclophosphamide and TBI at 400 cGy and post-transplantation Cyclophosphamide [56]. All patients were alive with a median follow-up of 705 days; one patient experienced graft failure; five patients reported aGVHD and 3 cGVHD, all with complete resolution; all patients, with the exception of one, were transfusion-independent post-HSCT [56].

Prior et al. compared the outcomes of allogeneic matched-related donor HSCT for SCD using four different conditioning regimens: anon-myeloablative regimen-based on TBI (300–400 cGy) + Sirolimus + ATG/Alemtuzumab [57]; Busulfan/Fludarabine + CNI/MTX + ATG/Alemtuzumab, as described by Krishnamurti et al. [58]; a non-myeloablative conditioning regimen based on Fludarabine/Melphalan + CNI/MTX + ATG/Alemtuzab, as described by King et al. [59]; and a myeloablative conditioning regimen based on Busulfan/Cyclophosphamide + CNI/MTX/Prednisone + ATG/Alemtuzumab as represented by Walters et al. [60]. For pediatric patients, the highest EFS was observed with the conditioning regimens reported by Krishnamurti et al. (3-year EFS of 94%) [58] and the lowest EFS with the non-myeloablative regimen of Hsieh et al. (3-year EFS of 57%) [22]. Rates of GVHD were lowest in the patients reported by Hsieh et al. (3-year rate of 0%) [22] and highest in the cohort of patients of King et al. (3-year rate of 20.4%) [53]. For adult patients, EFS rates were similar between the Hsieh et al. cohort [22] and the Krishnamurti et al. cohort [58], while cGVHD was lower in the Hsieh et al. cohort [22]. These findings suggest that pediatric patients had the highest EFS rates using myeloablative conditioning regimen, while non-myeloablative conditioning resulted in the lowest EFS rates; pediatric patients had the highest EFS rates using myeloablative conditioning regimens, while non-myeloablative conditioning regimens resulted in the lowest EFS rates [57].

Krishinamurti et al. have retrospectively analyzed a group of 763 SCD patients who have undergone allo-HSCT from MRD, HAPLO, MUD and MMUD donors after myeloablative, RIC or non-myeloablative conditioning and have analyzed in these patients the incidence and risk factors of pain crisis after HSCT [61]. The results of this study have shown that increased risk of pain crisis after HSCT was observed in patients aged >10 years at HSCT, in those aged ≥18 years, in those undergoing HSCT with alternate donors, either HAPLO, MUD or MMUD, and in those with graft failure [61].

Stenger et al. analyzed the long-term survival and late effects of allo-HSCT in a group of 1013 SCD patients undergoing allo-HSCT at 112 centers (51% MRD, 21% mismatch-related donors MMRD, 11% matched-unrelated donors and myeloablative in 66% of cases); GVHD prophylaxis was variable, involving tacrolimus, cyclosporine or post-transplant cyclophosphamide [62]. After a median follow-up of 60 months, 86% of transplanted patients remained cured with no SCD symptoms; most of patients had no SCD-related complications post-transplant; 70% of patients remained free of acute GVHD, and 74% of chronic GVHD [62]. At 1 and 7 years post-HSCT, OS and EFS were 95% and 90%, and 89% and 83%, respectively [62]. Older age at HSCT, alternative donor and grades III-IV GVHD were significantly associated with shorter survival [62]. Therefore, outcomes are superior in patients who undergo HSCT at a younger age, using BM MRD donors, and in those who remain GVHD-free [62].

### 3.5. Long-Term Effects of HSCT for SCD

The large number of HSCTs carried out in SCD patients allowed us to perform an analysis of the major determinants of the rate of success and of the complications (early and late) of this procedure. HSCT is a highly intensive treatment associated with many toxicities, which may impact patient-reported outcomes and the quality of life.

Recent studies, including increasing numbers of HSCTs with alternative donors, the introduction of reduced-intensity conditioning and non-myeloablative conditioning regimens and efficient GVHD prophylaxis, such as post-transplant Cyclophosphamide, have provided evidence that most SCD patients who underwent HSCT were alive, cured of SCD, and free of late events post-HSCT [63]. The meta-analysis of 58 studies of HSCT in SCD patients showed an OS rate of 94% and an EFS rate of 86% [32]. Clinical outcomes in these patients varied according to the type of donors, conditioning procedures/regimens, GVHD prophylaxis regimens and stem cell sources [32,63]. Older age at HSCT, alternative donors and grades III-IV aGVHD are determinants significantly associated with inferior survival [32,63].

Limited data defined long-term health outcomes after allo-HSCT for SCD, and, rarely, the HSCT outcomes were compared with chronic standard SCD therapies. In this context, some retrospective studies have analyzed long-term organ function after HSCT for SCD.

In SCD patients who received myeloablative HSCT from HLA-identical sibling donors with successful engraftment, there was the resolution of SCD-related events after transplantation, such as pain, stroke or acute chest pain [63]. Vaso-occlusive events represent key pathological determinants in the genesis of tissue damage. Vaso-occlusive events requiring medical care are significantly reduced in patients with SCD after allo-HSCT; this reduction improves over time and is particularly evident beyond 1-year post-HSCT [64].

SCD causes various central nervous system (CNS) abnormalities, and cerebral vasculopathy is the main indication for HSCT in SCD patients; usually, CNS abnormalities are stabilized or improved after HSCT, with normal or stable brain magnetic resonance imaging [65]. Cases of stroke after HSCT occurring in patients with pre-existing CNS disease and graft failure or rejection have been reported [63]. In adult SCD patients with CNS involvement with a history of depression, a high incidence of suicidal ideation was observed [66].

Cardiopulmonary complications are frequently observed in SCD patients and seem to stabilize or to improve 1–2 years after HSCT. However, more post-HSCT patients had lower cardiac ejection fraction than pre-HSCT patients [67].

At baseline, patients with SCD have to face some infertility risk factors, and this condition may be considerably impacted by gonadotoxic treatments associated with HSCT, caused by alkylating agents and TBI [32,62]. Conditioning with myeloablative Busulfan and Cyclophosphamide causes serious gonadotoxicity, particularly among post-pubertal females; reduced-intensity conditioning and non-myeloablative conditioning are less gonadotoxic, but their effects on fertility need to be assessed at long-term [62]. Female patients with SCD who undergo HSCT have high rates of diminished ovarian reserve, while the effects on premature ovarian insufficiency are variable [68]. These observations support the view that patients need to be counseled about the risk of infertility and informed about the strategies that can be adopted to favor fertility preservation.

An improvement in the quality of life of SCD patients who received allo-HSCT is a finding largely expected, given the curative effect of transplantation in these patients. However, it must be considered that HSCT is a highly intensive treatment associated with many toxicities, which might impact patient-reported outcomes and the quality of life. A prospective evaluation of patient-reported outcomes in 17 adolescents and adults with SCD undergoing HSCT showed a significant improvement in physical function and pain interference after 1 year [52]. Three other small retrospective studies have explored the effects of HSCT on quality of life, showing that patients with successful transplants are usually able to pursue their objectives, but fatigue and avascular necrosis can negatively affect the quality of life of some patients [69,70,71].

Two recent studies more carefully assessed the quality of life in SCD patients who received allo-HSCT. Nirmal et al. reported the assessment of patient-parent reported quality of life involving 62 SCD patients who underwent HSCT with a follow-up of 59 months post-transplant [72]. These patients experienced, post-HSCT, improved physical health, improved scholastic performance, decreased hospital visits and were able to pursue their personal life goals [72]. Dovern and coworkers explored changes in quality of life of adult SCD patients with SCD following allo-HSCT, through assessment of physical, mental and social health before HSCT and 6-, 12- and 18-months post-transplant [73]. The results of this assessment showed an improvement in quality of life at physical, mental and social health levels after HSCT; however, these effects on mental health are complex and support the need for psychosocial support early during the process of curative therapies [73].

## 4. Hematopoietic Stem Cell Transplantation in Beta-Thalassemia

HSCT is an established procedure in β-thalassemic patients with a consistent definition of the clinical and biologic parameters that affect transplantation outcomes.

### 4.1. HSCT in β-Thalassemic Patients with HLA-Identical Donors After a Myeloablative Conditioning Regimen

Studies carried out in the last 3–4 decades showed that HSCT is an important therapeutic option for β-thalassemic patients. The most consistent experience occurred in Pesaro, Italy, where a standard pre-transplantation risk assessment was developed. This system classifies β-thalassemic patients into three risk classes based on liver size assessed by physical examination, the presence or absence of liver fibrosis, as assessed by histological analysis of liver biopsy, and adherence to regular iron chelation: class I, no or minimal risk factors; class II, presence of one or two risk factors; and class III, presence of 3 risk factors [74,75]. For patients undergoing HSCT, assessment of liver pathology by liver biopsy plays a crucial role in risk stratification and clinical decision-making, particularly for patients displaying an abnormal liver elastography [76]. These studies showed that after HLA-matched sibling HSCT, the thalassemia-free survival probabilities for Pesaro class I or II and class III recipients were 87%, 85% and 80%, respectively [77,78]. Other studies outside Italy showed 5-year probabilities of OS and EFS for patients with Pesaro risk class II corresponding to 91% and 88%, respectively, and 64% and 62%, respectively, for Pesaro risk class III [79]. Mortality risks were higher for patients older than 7 years of age and those with hepatomegaly before HSCT [79].

**Figure 2:**
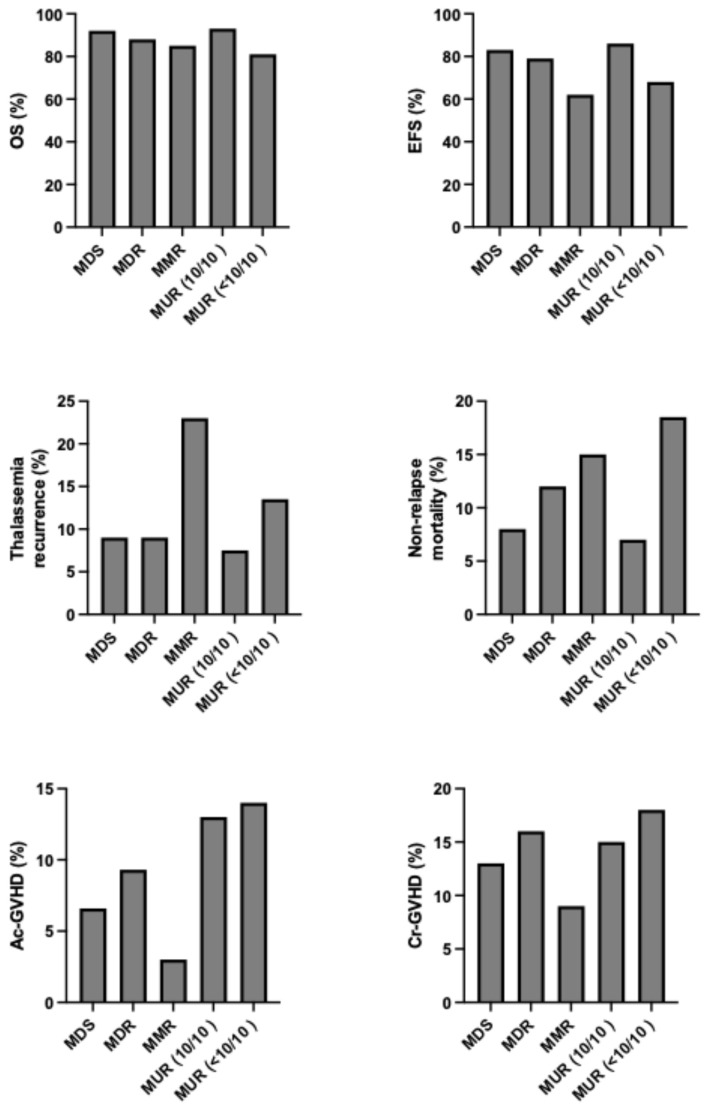
Outcomes of allo-HSCT in 2891 β-thalassemia patients reported in the EBMT registry. The patients have been subdivided according to the type of donor: MDS (matched donor sibling), MDR (matched donor relative), MMR (mismatch related), MUR 10/10 (matched unrelated with an HLA compatibility of 10/10), MUR < 10/10 (matched unrelated with a <10/10 HLA compatibility). Data are reported in [77].

A statistically significant difference in OS was reported in the group of patients with ages comprised between 10 and <14 years compared to those in the group between 14 and <18 years of age (96% and 82%, respectively); in the group of patients who are <18 years of age (96% vs. 82%, respectively); and in the group of patients who are >18 years, OS and TFS were 80% and 77%, respectively [80]. A subsequent EBMT study showed that an HSCT with a fully matched-unrelated donor is associated with outcomes identical to those obtained with an MSD (Figure 2) [10,81].

HSCT in β-thalassemia continuously expanded and the current worldwide experience showed OS rates around 90%, with disease-free survival around 70–80%; furthermore, advanced transplantation techniques, such as modified conditioning regimens, have reduced the risk for high-risk β-thalassemic patients, making outcomes more similar across all Pesaro classes; despite these improvements, factors like iron overload from pretreatment exposure negatively impact outcomes, particularly in older patients.

Recent retrospective analyses have provided a long-term evaluation of survival and of late effects of HSCT in β-thalassemia patients who have undergone allo-HSCT with a HLA-identical-related donor sibling after a myeloablative conditioning regimen [82]. In a group of 137 β-thalassemic patients who received allo-HSCT the cumulative incidence of nonrelapse mortality and thalassemia recurrence was 9.5% at 1 year and 10.2% at 39 years; the 39-years cumulative incidence of overall survival and disease-free survival were 81.4% and 74.5%, respectively; early death and late death transplant-related causes were observed in 9.5% and 0.7% of patients, respectively; full chimerism was observed in 94.5% of cured patients [82]. OS was related to the age at the moment of HSCT: 84.7% in patients < 10 years; 80.9% in patients 10–17 years; and 74.3% in patients > 18 years [82]. Patients cured had normal hematological parameters [82]. A total of 15 cases of secondary solid cancers were observed for a 39-year cumulative incidence of 16.4% [82]. The conclusion of this long-term evaluation of β-thalassemic patients who have received allo-HSCT was that allo-HSCT represents a curative option for most of these patients, at the price of a non-negligible early and late mortality [82]. Similar conclusions were reached by Caocci et al. in a long-term analysis on 258 β-thalassemic patients, including 97 adults (age ≥ 16 years) [83]. After a 30-year follow-up, OS among all patients was 82.6%, compared to 71.2% among adult patients [83].

The degree of iron overload and adequacy of its chelation are used for prognostic classification of thalassemia patients. Three iron-related factors associated with a significantly reduced probability of survival: hepatomegaly, hepatic portal fibrosis and inadequate iron chelation [77,78]. Several studies attempted to define a possible prognostic role of iron overload on allo-HSCT outcomes. Trottier et al., using liver magnetic resonance imaging to quantify liver iron content and serum ferritin levels to quantify systemic iron overload, found no association between iron overload and survival or complications in allo-HSCT recipients at 1-year post-transplant [84]. A more recent study confirmed these findings in a group of 152 children who received allo-HSCT for non-malignant hematologic conditions, showing that pre-transplant liver iron content and ferritin content were not significantly associated with poorer post-transplant outcomes [85]. The current treatment guideline for β-thalassemic patients undergoing allo-HSCT implies intensive post-transplant iron chelation (to reduce serum ferritin levels to below 1000 ng/mL or a liver iron concentration < 7 mg/g dry weight), temporary suspension during the acute transplant phase and post-transplant iron reduction therapy [86]. There is no strict indication for reinitiation of iron chelation therapy post-HSCT, which is usually re-initiated 5–50 months after transplant. The iron chelator deferasirox is increasingly used post-HSCT due to its better tolerance and efficacy in managing ongoing iron toxicity [87]. Magnetic resonance imaging assessment of the changes in cardiac and hepatic iron load in thalassemia patients before and after HSCT showed that there was no significant improvement in cardiac and liver iron content in thalassemia patients within 24 months after HSCT, while the reduction in cardiac and liver iron content in patients is evident when >24 months after HSCT [88].

**Figure 3:**
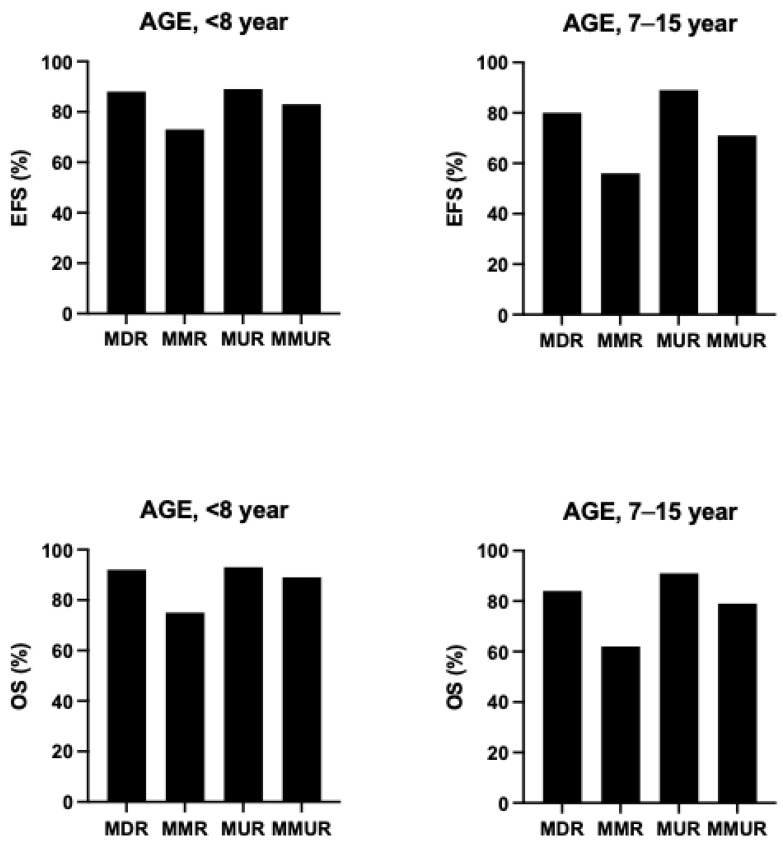
Event-free survival and overall survival in related and unrelated transplantation for β-thalassemia major. The patients were subdivided into two groups according to age at transplantation (<8 years and 7–15 years) and into four groups according to donor type: MRD, MMR, MUR and MMUR. Data reported in [89].

As discussed above, among the clinical determinants of transplantation outcomes, the most relevant is represented by the risk class of the patients, mainly defined by the absence or presence of hepatomegaly and portal hepatic fibrosis. An international survey no more than 1000 β-thalassemia patients undergoing HSCT showed that outcomes are strongly affected by the age of patients at the transplantation: overall survival for patients ≤ 6 years, 7 to 15 years, and 16 to 25 years, the 5-year OS was 90%, 84% and 63%, respectively and the EFS was 86%, 80% and 63%, respectively [89]. (Figure 3) Among the biologic factors, the most relevant determinant was HLA-matching: overall and event-free survival were similar for HLA-matched relative and HLA-matched-unrelated donor transplantation (OS 89% vs. 87% and EFS 86% vs. 82%, respectively); overall and event-free survival were lower for HLA-mismatched than HLA-matched transplants [89]. The graft failure for patients ≤ 6 years, 7 to 15 years and 16 to 25 years was 8%, 10% and 22%, respectively. A high rate of graft failure was observed after HLA-mismatched transplants (HLA-matched-unrelated 10.5% and HLA-mismatched relative 22%) [83]. aGVHD and cGVHD were comparable in HLA-mismatched relative and in HLA-mismatched unrelated 35% vs. 20% and 20% vs. 23%, respectively [89].

These findings were confirmed in a study of Xu et al. on 281 Chinese β-thalassemic patients who received HSCT from HLA-identical sibling donors; the patients were subdivided into three groups: group 1 was <4 years; group 2 was 5–8 years; and group 3 was >8 years. OS in groups 1, 2 and 3 were 96.2%, 95% and 85.2%, respectively [90]. These findings support the view that an age of >8 years at HSCT negatively affects transplantation outcomes [90].

In this context, it is important to note that HLA-typing techniques have led to the definition of five key human leukocyte antigen (HLA) loci (HLA-A, -B, -C, -DRB1 and -DQB1) crucial for immune recognition, whose matching between donor and recipient allows to define a condition of good compatibility (10/10 compatibility). Thus, although HLA-matched sibling transplantation represents the gold standard, an 8/8 allele-matched donor (at HLA-A, -B, and -C, and -DRB1) is the minimum requirement, but transplantation from 10/10 donors represents a good choice for patients without an HLA-matched donor. However, the achievement of 12/12 match across all six HLA loci, including HLA-DP1, is considered the gold standard for optimal outcomes, since mismatches are associated with a higher risk of GVHD and graft failure, particularly in HSCTs in patients with non-malignant conditions. In fact, HLA-DP1 mismatches in otherwise matched transplants associate with an increased incidence of GVHD [91] and post-transplant Cyclophosphamide improves survival in HLA-DP1 mismatched-unrelated donor allo-HSCT [92].

The availability of a matched donor sibling for HSCT is limited, so the probability of finding a suitable fully compatible donor is below 25%. Due to the paucity of matched donor availability, haploidentical HSCT (Haplo-HSCT) is a reasonable alternative. Two different regimens for haplo-HSCT were used: ex vivo T-cell depletion via TCRαβ/CD19^+^ depletion and in vivo T-cell depletion with post-transplantation cyclophosphamide.

### 4.2. Haplo-HSCT in β-Thalassemia Using Post-Transplant Cyclophosphamide as GVHD Prophylaxis

Post-transplant Cyclophosphamide is typically used in association with other immunosuppressive drugs, usually administered at days +3 and +4 post-transplant.

Wen et al. reported the study of 20 β-thalassemic patients (median age of 8 years) undergoing mismatched HSCT (15 unrelated and 5 related donors). The patients received a conditioning regimen containing thymoglobulin, Busulfan, Fludarabine and Thiotepa; GVHD prophylaxis consisted of Cyclophosphamide, Mycophenolate Mofetil and Thiotepa administered post-transplantation [93]. All transplanted patients had a successful engraftment with >95% of donor-derived cells at day 30 post-transplantation; after a median follow-up time of 38 months, OS and TFS were 100% [93]. The cumulative incidence of grades II-III acute GVHD was 10% (no patient developed grade IV GVHD); no patient developed either limited or extensive chronic GVHD [93]. The rates of OS, TFS and the incidence of acute and chronic GVHD compare favorably with those previously observed by the same authors in mismatched HSCT in β-thalassemic patients using a different strategy of GVHD prophylaxis [93]. Lu et al. reported the results observed in 40 β-thalassemic patients receiving allo-HSCT from 10 haploid-related donors (Haplo-RD) and from 30 unrelated donors, 15 with HLA-matched (MUD) and 15 with haploid HLA compatibility (Haplo-UD); these patients received HSCT after a conditioning regimen based on Fludarabine, Busulfan, Cyclophosphamide and Thiotepa and with a GVHD prophylaxis including post-transplantation Cyclophosphamide, ATG and Cyclosporine [94]. Importantly, in HSCT involving Haplo-RD and Haplo-UD, the GVHD regimen was based on a low-dose ATG (1 mg/kg/3 d) and a semi-dose of Cyclophosphamide (25 mg/kg/2 d) [94]. The incidence of grades III-IV acute GVHD was 7.5%, and of chronic GVHD was 10% [94]. The OS and TFS were both 92.5%. In the group of Haplo-RD patients, OS and TFS were both 100% [94]. Anurathapan and coworkers developed a reduced-intensity conditioning regimen (Fludarabine and Busulfan) and initially explored the regimen in HLA-matched allo-HSCT of β-thalassemic patients with age > 10 years and hepatomegaly, and observed that this regimen was comparable to a conventional myeloablative regimen in terms of OS and EFS [95]. In a subsequent study, this approach was extended to 83 β-thalassemic patients undergoing haplo-HSCT [96]. In this study, the patients were first submitted to a pharmacologic pretransplant immune suppression phase and two courses of dexamethasone and Fludarabine, followed by posttransplant conditioning with Fludarabine, i.v. Busulfan and post-transplant GVHD prophylaxis with Cyclophosphamide, Tacrolimus and mycophenolate mofetil [96]. The 3-year OS and EFS were both 96%, and there were no secondary graft failures [6]. A total of 7% of treated patients developed severe GVHD [96].

Hu et al. reported the results of a clinical study on 54 pediatric β-thalassemic patients who had undergone allo-HSCT from haploidentical donors after a myeloablative conditioning regimen, with post-transplant cyclophosphamide and low-dose methotrexate as a GVHD prophylaxis regimen [97]. After a median follow-up of 520 days, OS and EFS rates were 98.1% and 90.7%, respectively [97]. The cumulative incidence of grades II-III acute GVDH was 13.8%, and the frequency of chronic GVHD was 28.5% [97]. Importantly, in this study, it was shown that the inclusion of a high-dose cyclophosphamide dose (200 mg/Kg) compared to a low-dose cyclophosphamide dose (120 mg/Kg) resulted in a better OS and EFS and a lower incidence of chronic GVHD [97]. More recently, the same authors have reported the results of a retrospective analysis on 160 β-thalassemic patients undergoing allo-HSCT using PBSCs from haploidentical donors or matched-unrelated donors using the above-reported GVHD prophylaxis regimen based on post-transplant Cyclophosphamide [98]. The results of this study showed that OS, thalassemia-free survival, aGVHD and cGVHD were similar in HSCT from haploidentical and matched donors [98].

### 4.3. Haplo-HSCT in β-Thalassemia with TCRαβ^+^-Depleted Donor Cells

Ex vivo T and B-cell-depleted haplo-matched transplants represent another strategy for HSCT from haploidentical donors. T-cell and B-cell-depleted haplo-HSCT are associated with a low rate of acute and chronic GVHD and a low rate of transplant-related mortality, but with a high rate of graft failure; patients who experienced graft failure may be re-transplanted.

Several studies have explored the use of haploidentical TCR and B-cell-depleted donors in allo-HSCT in children with non-malignant disorders, including β-thalassemia. In this context, Bertaina and coworkers have explored in a clinical trial 23 children with non-malignant disorders who received HLA-haploidentical HSCT after ex vivo elimination of αβ^+^ T-cells and CD19^+^ B-cells. No patient received any post-transplantation pharmacologic prophylaxis of GVDH; all but four patients engrafted, but were rescued after a second transplant; transplantation-related mortality was 9.3%, and after 2 years, OS and DFS were both 91.1% [99].

Gaziev and coworkers have examined the outcomes of haplo-HSCT using TCRαβ^+^-depleted grafts in 14 children with hemoglobinopathies (TCR group) compared with the outcomes observed in a historical group of 40 patients with hemoglobinopathies transplanted with CD34^+^ selected grafts [100]. Patients received a conditioning regimen consisting of Busulfan, Thiotepa, Cyclophosphamide and ATG, preceded by Fludarabine, Hydroxyurea and Azathioprine. In the TCR group, the 5-year OS and DFS were 84% and 69%, respectively, with 14% of primary graft failure; in the CD34 group, the 5-year OS and DFS were 78% and 39%, respectively, with a graft failure of 45% [84]. The incidence of acute GVHD and chronic GVHD were 28% and 21% in the TCR group and 29% and 10% in the CD34 group [94]. Suboptimal recovery of CD4^+^ during the first six months post-transplantation was observed in both groups of patients [100].

Another clinical study reported the results observed in 70 pediatric patients with non-malignant disorders undergoing TCRαβ/CD19-depleted haploidentical HSCT from HLA-partially matched relative donors [101]. The median age at transplantation was 3.5 years; primary engraftment was observed in 51 patients, while 19 and 2 patients experience primary or secondary graft failure, respectively; most graft failures were observed in children with aplastic anemia or β-thalassemia; all but 5 patients exhibiting graft failure were retransplanted; 6 patients (8.5%) died of infectious complications; grades I or II GVHD was observed in 14.4% of patients; and the 5-year probability of overall and disease-free survival were 91.4% and 86.8%, respectively [101].

Since a complete ex vivo T-cell depletion while mitigating the risk of developing GVHD, exposes to a higher risk of graft failure and of severe infections due to delayed immune reconstitution, a strategy was developed for CD3^+^/CD19^+^ depletion with a targeted CD3^+^ addback of 1 × 10^5^ CD3^+^ T cells/Kg to the graft before infusion [96]. All 12 patients displayed rapid engraftment and were alive with >92% of peripheral blood chimerism [102]. No patient developed grades III-IV GVHD [102]. Another study confirmed the safety and efficacy of unrelated donor stem cell transplantation with partial T-cell depletion in a group of 16 pediatric patients with hemoglobinopathies [103].

Liao and coworkers have reported a study on 266 β-thalassemic patients undergoing haplo-HSCT with TCRαβ^+^-depleted donor cells with the specific aim of defining an optimal conditioning regimen for HSC engraftment [104]. The patients were subdivided into two groups depending on the timing of ATG and Cyclophosphamide administration.

In the TDH-A group, ATG was administered on days −8 to −7 without Cyclophosphamide on days −2 and −1; in the TDH-B group, ATG was administered on days −21 to −19, with 50 mg/Kg cyclophosphamide on day −2 and 25 mg/Kg cyclophosphamide on day −1 [105]. The rest of the conditioning regimen was identical for the two groups of patients and was based on cyclophosphamide, Busulfan, Fludarabine and Thiotepa; the conditioning regimen also included the infusion of a low number of donor lymphocytes [105]. Remarkable differences were observed for the TDH-A and TDH-B groups. In the TDH-A group, 30 patients were enrolled, 18 (60%) achieved a successful engraftment, while 12 (40%) had primary graft failure (all underwent salvage HSCT with alternative donors); one death occurred and with a follow-up of 1523 days, and OS, TFS, TRM (transplant-related mortality) were 96.7%, 56.7% and 40%, respectively [105]. In the group TDH-B, 236 β-thalassemic patients were enrolled, 231 achieving a successful engraftment (97.9%), while (2.1%) showing primary graft failure; 10 deaths occurred, and with a follow-up of 717 days, OS. TFS and TRM were 95.4%, 94.1% and 4.6%, respectively [105]. Grades III-IV GVHD was 10.5% among TDH-A and 4.7% among TDH-B patients; chronic GVHD was 16.6% in the TDH-A group and 8.6% in the TDH-B group [105]. These observations showed that advancing ATG administration to day −21 (group TDH-B) significantly promoted engraftment; pretransplant (days −2/−1) administration of Cyclophosphamide clears recipient lymphocytes without compromising donor stem cell engraftment with low rates of acute and chronic GVHD [105].

Giardino and coworkers have retrospectively analyzed 20 children with congenital non-malignant disease who underwent haploidentical stem cell transplantation after TCRαβ^+^ and CD19^+^ depletion; no GVHD prophylaxis was given when TCRαβ^+^ cells in the graft were <1 × 10^5^ cells/Kg [106]. Engraftment was observed in 85% of patients; 15% of patients displayed primary graft failure; and 10% of patients exhibited secondary graft failure; all these patients received a second transplant [106]. The cumulative incidence of acute and chronic GVHD was 15% [106]. After a median follow-up of 4 years, 18 patients (90%) were alive [106].

In a group of 196 β-thalassemic patients who have undergone allo-HSCT, Liao et al. have analyzed the effect of different stem cells used for transplantation on the immune reconstitution, GVHD and viral infections. Three groups of HSCTs were analyzed: MUD-HSCT, Haplo-HSCT and TCRαβ^+^ T-cell-depleted HSCT (TD-HSCT) [107]. TD-HSCTs exhibited a faster immune reconstitution compared to the other two groups; TDH and MUD-HSCTs were associated with a lower incidence of cGVHD; and the occurrence of viral infections was higher in the TDH group than in the other two groups [107].

Meissner et al. have retrospectively analyzed 124 transfusion-dependent β-thalassemic patients, Pesaro I–II risk class, who have undergone allo-HSCT. OS and TFS were 94.5% and 88% after Threosulfan-Fludarabine-Thiotepa and 96.9% and 96.9% after Busulfan-Fludarabine-based conditioning; mixed chimerism below 75% occurred predominantly in Treosulfan-based regimens; OS and TFS did not differ significantly between various types of matched donors; mismatched UD-HSCTs displayed a lower OS and TFS rate [108]. The main risk-reducing factors were represented by high CD3^+^ cell count in the graft, Busulfan-conditioning, and pre-conditioning therapy [108].

### 4.4. Allo-HSCT in β-Thalassemic Patients with Reduced-Intensity Conditioning Regimens

Patient characteristics, such as age, Pesaro risk class, and availability of suitable donors, are key determinant factors for a successful HSCT in β-thalassemia patients. Over the last few years, conditioning regimens for allo-HSCT of β-thalassemic patients have evolved to provide an adaptation to overcome patient-related limitations, to increase the opportunity of transplantation for more patients, including unrelated donors, and to reduce adverse outcomes related to toxicity events or to graft failure or GVHD [109].

Several studies have explored the use of cytotoxic agents less toxic than Busulfan in the conditioning regimen. Treosulfan (dihydroxybusulfan), structurally similar to Busulfan, is a prodrug alternative to Busulfan conditioning prior to HSCT. Treosulfan is administered as a prodrug and is converted nonenzymatically, generating various active metabolites that are responsible for its cytotoxic effects.

The standard myeloablative conditioning used for allo-HSCT of β-thalassemic patients consisted of Busulfan (14–16 mg/Kg), Cyclophosphamide (160–200 mg/Kg) with or without ATG. In high-risk β-thalassemic patients is associated with a significant toxicity and a significant rate of graft rejection. The introduction of a toxicity-reduced conditioning regimen containing Treosulfan, Fludabarine and Thiotepa and a peripheral blood stem cell graft in older β-thalassemic patients with high-risk disease (Pesaro class III) resulted in improved transplant outcomes (5-year OS 71.9% with Treosulfan compared to 54.5% with Busulfan) and a reduction in early transplant-related mortality (46% with Busulfan and 13% with Treosulfan [110,111].

The dose exposure-response studies suggest a consistent interindividual variation in Treosulfan exposure resulting in variable HSCT outcomes, supporting the importance of drug monitoring of Treosulfan [112]. Measuring the pharmacokinetics of Treosulfan at the first dose and individualizing the third dose may represent an optimal strategy in non-malignant diseases [112]. Van der Stoep et al. reported the study of 110 pediatric patients undergoing allo-HSCT after a Treosulfan-based conditioning regimen; the analysis of the exposure to Treosulfan of these patients showed a relationship with early and long-term clinical outcomes [113]. A similar study in β-thalassemia patients who underwent allo-HSCT after a Treosulfan-based conditioning regimen showed that exposure to this drug predicts thalassemia-free survival [114]. Importantly, lower Treosulfan exposure resulted in an increased risk of graft rejection and early transplant-related mortality [114].

A large retrospective study (772 β-thalassemic patients, 410 treated with a Busulfan regimen and 362 with Treosulfan regimen) showed that the use of Busulfan or Treosulfan as the backbone of myeloablative conditioning for patients with transfusion-dependent thalassemia undergoing HSCT resulted in comparable high cure rates (2-year OS of 92.7% after Busulfan and Fludarabine conditioning and 94.7% after Treosulfan and Fludarabine conditioning) [115]. Sykora et al. have reported the results of a prospective randomized, phase II trial comparing the safety and the efficacy of Busulfan plus Fludarabine with Treosulfan plus Fludarabine preparative regimens in patients with non-malignant disease undergoing allo-HSCT [116]. Treosulfan-based regimen resulted in less toxicity than Busulfan, showing a lower transplantation-related mortality than Busulfan (3.9% vs. 12%, respectively) [116]. Overall survival favored Treosulfan (96%) compared to Busulfan (88%) [116]. The rate of graft failure, particularly of secondary graft failure, was higher in transplantations conditioned with Treosulfan compared to Busulfan [116]. Acute GVHD of at least grades I/II was observed in 8% in the Busulfan arm as compared to 13.7% in the Treosulfan arm. Moderate/severe chronic GVHD was more frequent among patients treated with Busulfan (14%) compared to Treosulfan (2%) [116].

Kleinschmidt and coworkers have explored the safety and efficacy of T-cell-depleted haplo-HSCT for pediatric and young adult β-thalassemic patients [117]. Twenty patients with transfusion-dependent thalassemia (median age 10 years) received either a matched sibling donor or a haplo-HSCT after a conditioning regimen based on ATG, Treosulfan, Thiotepa and Fludarabine; immunosuppression consisted of calcineurin inhibitor and mycophenolate mofetil [117]. At a median follow-up of 37 months, OS and DFS were both 100% for MDS and 92% in haplo-HSCT; two patients in the haplo-HSCT had graft failure; and no acute GVHD > grade III or severe chronic GVHD were observed [117].

Treosulfan is generally associated with a favorable long-term safety profile and fewer toxicities compared to Busulfan. Long-term follow-up studies in children with β-thalassemia post-HSCT show no significant reduction in growth potential, with up to 80% of patients achieving optimal growth rate [118]. Treosulfan-based regimens are associated with low endothelial toxicity and less passage across the blood–brain barrier compared to Busulfan [119]. Although most of the acute toxicities and long-term adverse effects are less frequent in SCD and β-thalassemia patients who underwent allo-HSCT with a Treosulfan-based regimen compared to a Busulfan-based regimen, it was noted that higher Treosulfan exposure increases the risk of skin toxicity [113].

## 5. Different Sources of HSCs for HSCT in SCD and β-Thalassemic Patients

Bone marrow (BM), peripheral blood (PB) and cord blood (CB) represent a suitable source of HSCs for HSCT in SCD and β-thalassemic patients. Bone marrow transplantation (BMT), peripheral blood stem cell transplantation (PBSCT) and cord blood stem cell transplantation (CBSCT) represent effective, potentially curative options for SCD and β-thalassemia, usually associated with comparable survival outcomes.

Few studies have directly compared the outcomes of PBSCT and BMT in β-thalassemic patients. Studies carried out using these two different sources of HSCs showed that both are safe for donors, being associated with a low rate of adverse events. PBSCs induced a faster platelet and neutrophil recovery after HSCT, probably related to a higher content of CD34^+^ cells in mobilized PB than in BM, and PBSCT is usually associated with a higher risk of developing GVHD than BMT. In a first study, Ghavamzadeh et al. compared classes I-II Pesaro-risk β-thalassemic children who underwent HLA-matched PBSCT or BMT after a myeloablative conditioning regimen based on Busulfan and Cyclophosphamide, followed by Cyclosporine +/− Methotrexate for GVHD prophylaxis [120]. The comparative analysis of the outcomes observed in 87 PBSCT patients and 96 BMT patients showed that: the median time to neutrophil and platelet recovery in PBSCT was significantly shorter than in BMT patients; grades II-IV GVHD and cGVHD were more frequent in PBSCT than in the BMT group; and the 2-year OS was similar after PBSCT and BMT (83% vs. 89%, respectively) [120]. A second, more recent study by the same authors, based on the long-term outcomes of 567 β-thalassemic patients who received allo-HSCT between 1998 and 2015, showed a similar OS after BMT and PBSCT; aGVHD and cGVHD were more frequent after PBSCT compared to after BMT, but the difference was not statistically significant [121].

Locatelli et al. analyzed the outcomes of 485 patients with β-thalassemia major or SCD who received HLA-identical CBSCT or BMT [122]. In comparison with patients receiving BMT, those receiving CBSCT had slower neutrophil recovery, less aGVHD, and no extensive cGVHD [122]. The 6-year OS after BMT and CBSCT was 95% and 97%; the 6-year DFS was 86% and 80% in β-thalassemia patients after BMT and CBSCT, respectively, and 92% and 90% in SCD patients after BMT and CBSCT, respectively [122].

The analysis of 44 patients with β-thalassemia or SCD who underwent related CBSCT with a GVHD prophylaxis based on Cyclosporin A alone or in association with methotrexate showed that 36 patients displayed engraftment and 8 patients a graft failure (6 primary and 2 secondary graft failure); 3 of these 8 patients had sustained donor engraftment after BMT from the same donor [123]. The occurrence of GVHD was low, with 4 patients experiencing grade II aGVHD and two limited cGVHD [123].

Zwolsman and coworkers have retrospectively evaluated 44 SCD patients undergoing haploidentical HSCT: 26 patients received BMT, and 15 received PBSCT [119]. All the patients received a non-myeloablative conditioning regimen consisting of ATG, Thiotepa, Fludarabine, Cyclophosphamide, TBI (2Gy) and post-transplantation Cyclophosphamide. The 1-year EFS was 100% in the PBSCT group and 85% in the BM group [124]. Initially, all patients engrafted; one BM recipient displayed secondary growth failure with autologous reconstitution; three patients in the BMT group died [119]. Future prospective studies are required to compare haplo-HSCT in SCD patients using BM or PBSC [124].

Two recent studies explored the safety and efficacy of PBSCs for haplo-HSCT in adult patients with severe SCD [40,125]. These studies reported the outcomes observed in 22 consecutive adults with severe SCD who underwent haplo-HSCT with PBSCs after a conditioning regimen based on ATG, Fludarabine, Cyclophosphamide, TBI (3Gy) and post-transplantation Cyclophosphamide [45,125]. The patients received an infusion of G-CSF-mobilized haploidentical PBSCs [45,125]. After a follow-up of 4.8 years, 95% of patients were alive, 86% were alive without GVHD, 90% remained free of a vascular occlusive event; Hb levels increased from 8.2 g/dL pre-transplantation to 14.3 g/dL post-transplantation; a GVHD grade of ≥2 was observed in two patients; and moderate to severe cGVHD occurred in three patients [45]. Organ damage/dysfunction remained stable or improved [45,125].

## 6. Complications Associated with Allo-HSCT in Patients with Hemoglobinopathies

Allo-HSCT in β-thalassemic and SCD patients is frequently associated with some complications that can impair the outcomes of transplantation in these patients.

### 6.1. Acute and Chronic GVHD

GVHD represents one of the most frequent complications after allo-HSCT, particularly in patients undergoing mismatched HSCT. In a recent systematic review and meta-analysis of allo-HSCT studies in SCD patients, a global incidence of 20% of acute-GVHD (aGVHD) and 14% of chronic GVHD (cGVHD) was estimated [32]. In a recent analysis of outcomes and complications observed in β-thalassemia patients who received allo-HSCT in the United States, a frequency of aGVHD of 42% and of cGVHD of 24% was reported [126]. The analysis of the EBMT registry, including 2807 consecutive β-thalassemia patients who underwent allo-HSCT (mostly from MRDs), showed that the rate of aGVHD grades III/IV and cGVHD extensive occurred in 8.4% and 4.4% of children and 9.5% and 7.2% of adults, respectively [127].

GVHD results from immune reactions triggered by dissimilar HLA antigen molecules, inducing the immune activation of donor T-lymphocytes. Unrelated HSCT or partially HLA-mismatched HSCT are associated with an increased risk of severe GVHD, graft failure and non-relapse mortality. Strategies to prevent and to mitigate GVHD are essential to ensure the results of allo-HSCT. GVHD prophylaxis was achieved using different agents, such as calcineurin inhibitor (CNI) combined with methotrexate (MTX), anti-lymphocyte antibodies polyclonal (anti-thymocyte globulin, ATG), or monoclonal (Alemtuzumab, an antibody targeting CD52, a cell surface antigen found on most lymphocytes, NK cells and monocytes) or post-transplant Cyclophosphamide. Most of these treatments showed a consistent efficacy in preventing GVHD in alloHSCT in SCD and β-thalassemia patients.

Recently, it was introduced in the therapy Abatacept (a recombinant fusion protein consisting of the extracellular domain of human CTLA-4 connected to the Fc of human IgG1), which inhibits antibody-dependent, cell-mediated cytotoxicity and/or complement fixation. Experimental, preclinical studies have supported the clinical use of Abatacept in GVHD prophylaxis in SCD and β-thalassemia patients undergoing allo-HSCT.

Thus, Ngwube and coworkers showed that the addition of Abatacept to a standard GVHD prophylaxis regimen (Tacrolimus and MTX) resulted in a low frequency of GVHD in SCD patients undergoing one-antigen-mismatched-unrelated donor HSCT, after reduced-intensity conditioning [128]. Thus, the incidence of GVHD grades II to IV and grades III to IV at day +100 was 28% and 7%, respectively [128]. One-year incidence of cGVHD was 57% and mild/limited in all cases but one, for a patient who received Abatacept for a longer duration. The 2-year OS was 100%, and DFS was 92.9% [123]. In a more recent study, the same authors evaluated the impact of Abatacept incorporated into prednisone-including GVHD prophylaxis compared to standard prophylaxis without Abatacept, in pediatric patients with hemoglobinopathies undergoing allo-HSCT from related or unrelated donors after reduced-intensity conditioning [129]. The incidence of posterior reversible encephalopathy syndrome was 17% with standard GVHD prophylaxis and 0% with Abatacept [129]. Acute grades 3 to 4 GVHD occurred in 28% of patients who received standard GVHD prophylaxis and 0% of patients treated with Abatacept. Among patients who received standard GVHD prophylaxis, cGVHD was observed in 2.5% (mild), 5% (moderate) and 30% (severe) of cases, compared to 25% (mild), 5% (moderate) and 5% (severe) in those treated with Abatacept [129]. OS and EFS were 87% and 80% in patients who had received standard GVHD prophylaxis and 95% and 90%, respectively, in patients treated with Abatacept [129].

Khandelwal et al. showed that GVHD prophylaxis with Abatacept reduced severe GVHD in allo-HSCT for β-thalassemia after a myeloablative conditioned regimen with Busulfan, Fludarabine and Thiotepa. In particular, the addition of Abatacept to a standard GVHD prophylaxis regimen with CNI and methylprednisolone reduced to 0% the occurrence of grades III-IV GVHD at day +100 compared to a frequency of 50% observed in patients who underwent standard GVHD prophylaxis without Abatacept [130]. Thalassemia-free survival after HSCT was 100% in the Abatacept cohort compared to 62.5% in the standard cohort [130].

Jaiswal et al. reported the development of a GVHD prophylaxis protocol in patients with non-malignant diseases undergoing haplo-HSCT from haploidentical familial donors, based on Abatacept administration on days 0, +5, +20, and +35, and every 28 days thereafter until day +180, post-transplantation Cyclophosphamide, administered on day +3 and +4, and Sirolimus, given from day −7 to 9 months post-HSCT [131]. This GVHD prophylaxis protocol was explored in 40 patients with non-malignant diseases (24 with hemoglobinopathies), showing the following results: post-transplantation hemophagocytic syndrome was detected in 3 patients, leading to graft failure in 2 cases; the incidence of aGVHD was 2.6%, and that of cGVHD was 14.3%; rates of non-relapse mortality, overall survival, event-free survival, and GVHD-free, event-free survival were 5%, 95%, 90% and 82%, respectively [132]. The absence of cGVHD correlated with younger patient age [132].

Dexter and coworkers compared the outcomes observed in 35 consecutive patients with hemoglobinopathies who underwent allo-HSCT and received Abatacept GVHD prophylaxis to those observed in a historical group of 66 patients who received allo-HSCT after an identical conditioning regimen and with a GVHD prophylaxis without Abatacept [133]. All patients in the Abatacept group engrafted and are alive, whereas there were two cases of graft failure (3%) and three deaths (4.5%) in the control group [133].

### 6.2. Graft Failure and Second Transplantation

Graft failure is a critical complication of allo-HSCT for SCD and β-thalassemia. Graft failure can be related to a wide range of possibilities, including cell dosing, disease, infection, drugs and an immune-mediated event; in contrast, graft rejection is a process related only to an immune-mediated mechanism [134]. Graft failure may be classified as primary graft, defined as a lack of achievement of absolute neutrophil count (ANC) ≥ 500/µL by day +30 with associated pancytopenia, and secondary graft failure, defined as a decline in hematopoietic function necessitating blood products or growth factor support after having reached the standard levels of hematopoietic (neutrophil and platelet) recovery [134].

Some risk factors contribute to graft failure in patients with beta-hemoglobinopathies undergoing allo-HSCT, mainly related to hyperactive marrow and allo-sensitization, conditioning intensity, donor type, and iron overload.

The condition of chronic hemolytic anemia observed in both SCD and β-thalassemia creates a condition of marrow hypercellularity that may reduce the possibilities of engraftment of donor HSCs.

Many patients with SCD and β-thalassemia may be allo-sensitized due to chronic blood transfusion, increasing the risk of immune-mediated rejection. Thus, a retrospective study on 229 pediatric patients who received HLA-matched-related donor HSCT with myeloablative or reduced-intensity regimens showed that 17% of these patients display RBC alloimmunization pre-HSCT and more frequently experience graft failure (10% vs. 2.6%) and grades III-IV GVHD (15% vs. 3.7%) more frequently compared to the rest of the patients without anti-RBC alloimmunization [135]. Other studies suggest a possible link between anti-HLA antibodies prior to HSCT and the development of graft failure and delayed platelet recovery post-HSCT [136].

As discussed above, the use of reduced-intensity regimens minimizes toxicity but carries a higher risk of graft rejection compared to myeloablative conditioning. The use of alternative donors, such as haploidentical donors or unrelated donors, is associated with higher failure rates compared to matched sibling donors.

Finally, a condition of iron overload is observed in most patients with beta-hemoglobinopathies as a consequence of chronic transfusion, and creates an oxidative microenvironment in the bone marrow that may impair donor cell engraftment and differentiation.

A recent study retrospectively evaluated 1858 SCD transplant recipients, including 323 patients who experienced graft failure: 266 patients experienced graft failure ≤ 24 months post-HSCT and 57 ≥ 24 months post-HSCT [137]. Early graft failure occurred at a median of 3.7 months, as opposed to late graft failure, which occurred at a median of 35 months post-HSCT [137]. The analysis of the whole population of patients exhibiting graft failure occurred more frequently in patients with high mismatching (<8/8 HLA matching), in patients who received alternate donor HSCT, in patients transplanted with PBSCs, in patients who received non-myeloablative conditioning, and in patients with a history of stroke prior to HSCT [137]. Differences in patient demographics, donor type, graft type, HLA match, and GVHD prophylaxis were unable to distinguish early from late graft failures. Non-myeloablative conditioning regimen is significantly associated with higher odds of late graft failure [137]. Surprisingly, late graft failure was less frequent in patients with HLA-mismatched donors.

Recurrence of SCD or of β-thalassemia with autologous marrow recovery usually follows graft failure; however, in a significant proportion of cases, marrow aplasia may occur.

A second HSCT for SCD or β-thalassemic patients is a salvage option for patients who have experienced a primary or secondary graft failure after the first transplant, using the same or new donors, often requiring a stronger conditioning regimen, with success related to some factors, including timing after the first transplant and after graft failure, donor match, and patient health.

Several studies have reported the outcomes observed in the second HSCT in β-thalassemia patients. A total of 16 patients who experienced graft failure after a first HSCT (mostly primary graft failure) received a second HSCT with a median interval of 28 months between the two transplants [138]. The treatment protocol of the second transplantation was complex, involving a preconditioning phase with Hydroxyurea daily from −45 pretransplant, Fludarabine from day 16 to day 12, followed by conditioning with Busulfan, Thiotepa, Cyclophosphamide and ATG; the patients received pre-transplant iron chelation with Deferoxamine and RBC transfusions [138]. GVHD prophylaxis consisted of Cyclophosphamide post-transplant, Methotrexate and Cyclosporin A [138]. Overall survival rate, EFS rate, transplant-related mortality rate and graft failure rate were 79%, 79%, 16% and 6%, respectively [138]. A total of 13/16 patients were alive, and 3/16 patients died from complications related to transplantation [138]. Using this transplantation protocol, the same authors more recently reported the outcomes of 21 β-thalassemic patients who received a second HSCT after graft failure (10 primary graft failure, 11 graft failure); median time to second HSCT was 41 months [139]. All but 4 received a second HSCT from the same donor; 13 received BM and 8 received PB [139]. The incidence of aGVHD was 50% among patients who received PB and 8% in patients receiving BM; moderate/severe cGVHD was markedly more frequent among patients receiving PB compared to BM (93% vs. 0%, respectively). A total of 18/21 patients were alive with a median follow-up of 10 years [139]. This study also showed that the intensified treatment protocol was not associated with increased nonhematological toxicities [139].

Stepensky et al. in 2009 reported an unfavorable outcome in nine β-thalassemic patients who received a second HSCT after a graft failure in a first HSCT; these patients received different RIC regimens [140]. Following the second HSCT, 33% of patients had early mortality, 66% developed GVHD, and only 40% were transfusion-independent [140].

Korula et al. retrospectively analyzed 62 patients with graft failure after a first allo-HSCT: 52% primary graft failure (15 with marrow aplasia and 17 with autologous recovery) and 48% secondary graft failure (5 with marrow aplasia and 25 with autologous recovery); 46% of these patients underwent second HSCT and, with the exception of 1 patient, the donor for the second transplant was the donor used in the first HSCT [141]. Conditioning regimen for second HSCT was Busulfan-based MAC in 24% of patients, Treosulfan-based MAC in 41% of patients, and different non-myeloablative regimens in 35% of patients [141]. The best clinical outcomes were observed in patients who received Treosulfan-based MAC [141].

Recently, Al-Jafri et al. retrospectively analyzed the outcomes of 15 β-thalassemic patients who received a second HSCT after graft failure observed after the first HSCT [142]. In these patients, graft failure occurred over a median of 8.6 months after the first HSCT; the median time of the second transplant was 25.3 months, and all patients received the same donor used in the first HSCT [142]. For the second HSCT, the patients received a conditioning regimen based on TBI (1200 cGy subdivided into six doses over 3 days) and Cyclophosphamide [142]. A total of 86% of patients were engrafted, with a thalassemia-free survival of 80%; one patient rejected the graft and died; and one patient died due to infectious complications [142].

Ma and coworkers recently reported a retrospective analysis of 272 β-thalassemia patients from 18 Chinese centers who underwent second allo-HSCT due to graft failure of the first transplant; 71% of these patients had primary graft failure; 70.6% of these patients used a different second donor [143]. Neutrophil engraftment was achieved in 86.3% of patients by day 28 and in 90.6% by day 65; platelet engraftment was achieved in 70% of patients by day 100 [143]. The 3-year cumulative incidence of GVHD-, cGVHD-, relapse- and transplantation-related mortality were 43.5%, 27.8%, 15.6% and 49.5%, respectively; the 1-year and 3-year OS were 56.1 and 49.5%, respectively. The comparison of the outcomes observed using different donors and the same donor showed that changing donors significantly improved neutrophil (92.4% vs. 71.4%) and platelet engraftment (76.9% vs. 51.8%), 1-year TRM (34.8% vs. 56.3%) and OS (61.9% vs. 42.7%) [143]. The benefit of donor changing was limited to patients with primary graft failure [143].

SCD and β-thalassemia patients who experienced a failure of a first HSCT have two possible curative therapies: a second HSCT or a gene therapy [144]. However, demonstration of the feasibility or safety of HSC-based gene therapy after failed allo-HSCT has not been achieved and may be evaluated at the level of individual patients on a case-by-case basis, taking into account various factors related to the patient and HSC that may influence feasibility [144].

### 6.3. Hematologic Malignancies Post-Transplantation in SCD and β-Thalassemic Patients

Population studies have reported an increased risk of hematologic malignancies in patients with SCD compared with the general population [145,146]. Particularly, SCD patients aged 15 to 39 years were more likely to develop leukemia compared to those under 15 years [146]. Furthermore, patients with more severe disease were more likely to develop leukemia compared to those with less severe disease [146].

Various studies have explored the occurrence of secondary neoplasms after HSCT for SCD. Eapen et al. explored a cohort of 1096 transplants for SCD; the 10-year incidence of any secondary neoplasms was 2.4% and 1.7% of leukemia/MDS [147]. The incidence of secondary neoplasms was higher with low-intensity regimens (non-myeloablative), including total-body irradiation, compared to myeloablative regimens [147]. Lawal explored 120 patients with SCD who underwent allo-HSCT and reported that 8 of these patients developed hematologic malignancies between 4 months and 9 years post-HSCT; importantly, these 8 patients displayed either a graft failure or a condition of mixed chimerism [148]. Two of these eight patients had the same *TP53* mutations at baseline [149]. These authors, using an ultradeep sequencing method, showed pre-existing *TP53* mutations with a VAF ranging from 0.06% to 0.34% in four SCD patients developing a myeloid neoplasia after allo-SCT [150]. In line with these findings, Ali and coworkers showed that recipient cells and not donor cells are the source of hematologic malignancies after graft failure and mixed chimerism in SCD patients [151]. Gondek et al. have proposed a theory to explain why, in SCD patients, a curative therapy based on allo-HSCT may favor the development of leukemia. Individuals with SCD may develop clonal hematopoiesis that usually does not expand and does not progress to leukemia over time. However, after graft failure, the pressure of changing from homeostatic to regenerative hematopoiesis may drive the clonal expansion and leukemic transformation of pre-existing premalignant clones, inducing in some patients the development of leukemia [152].

After allo-HSCT, a small number of donor HSCs reconstitute the recipient hematopoietic system, whereas the donor remains with a near-normal pool of HSCs [136]. This conclusion was reached through genome sequencing of donor–recipient pairs taken many years after HLA-matched sibling HSCT: with young donors, about 5000–30,000 HSCs had engrafted and contributed long-term to hematopoiesis; with older donors, about 10-fold fewer HSCs stably engrafted [153].

Leukemia development has been associated with the acquisition of somatic mutations, which accumulate with aging in a condition defined as clonal hematopoiesis (CH). Somatic mutations in genes involved in stem cell self-renewal and differentiation are present in a minority of stem/progenitor cells and confer a survival or proliferation advantage, giving rise to the development of a small clone, resulting in its significant expansion compared with non-mutant cells. Whole exome sequencing studies on 1459 patients with SCD and 6848 African American controls have shown that SCD is associated with an increased prevalence of CH, with more frequent mutations on *DNMT3A* and *TP53* genes [154]. *TP53*, which is infrequently affected in the healthy population, was the second most frequently mutated gene after *DNMT3A* and accounted for 13% of all CH mutations in SCD patients [137]. In this study, it was postulated that ineffective erythropoiesis and increased erythrocyte turnover may favor the development of a clone carrying a selective growth advantage; an additional mechanism could be related to chronic inflammation [154].

These observations suggest that SCD itself may predispose patients to high-risk CH. Weeks et al. have screened a large cohort of SCD patients, normal individuals and patients with other hemoglobinopathies and observed that: CH occurred earlier in SCD patients and was more prevalent in SCD cases compared to non-SCD controls among individuals aged 0–19 years (10.6% vs. 3.5%, respectively) [155]. This difference was selectively driven by mutations of DNA damage response (DDR) driver genes (*TP53, PPM1D, CHEK2, ATM*) [155]. Prevalence of *DNMT3A* and *TET2* mutations was higher in SCD among the youngest population than in normal controls age-matched (6.4% vs. 2.5%, respectively) but progressively decreased with advancing age [155]. This precocious DDR-CH is specific to SCD and is not observed in individuals with sickle cell trait [155]. Given these observations, De Luna and coworkers have screened the occurrence of CH in 56 SCD patients candidates to transplant approaches and observed that 11 of these patients display CH mutations, the most frequent being *DNMT3A* (29%), *TP53* (29%), *PPMD1* (29%), and *ASXL1* (21%) [156]. The presence of high-risk mutations, such as *TP53*, in these patients supports the inclusion of molecular profiling in the context of pre-transplant evaluation [156].

However, it must be pointed out that characterization of clonal dynamics using ultradeep duplex sequencing in donor–recipient pairs decades after HSCT indicates that usually the evolution of CH is not accelerated as a result of HSC [157].

Several studies have explored the occurrence of solid cancers and hematologic malignancies in β-thalassemic patients who received allo-HSCT. The analysis of risk of cancer in β-thalassemic patients showed a significantly increased risk of developing cancer, particularly hepatocellular carcinoma, compared to both β-thalassemic trait subjects and the control general population [158].

Santarone et al. have reported the long-term occurrence of secondary cancers in β-thalassemic patients who received allo-HSCT [159]. With a follow-up of 39 years, the cumulative incidence of developing solid cancers in 122 transplanted β-thalassemic patients was 13.2%, compared to 3.2% and 1.3% in a population of age-matched HSC donors or in a population of β-thalassemic patients who received conventional nontransplant therapy, respectively [159]. In another study, the same authors reported a 39-year cumulative incidence of secondary solid cancer at 16.4%, with only one case of non-Hodgkin lymphoma reported as a hematologic malignancy among secondary cancers [82].

Solomou et al. reported a high incidence (15.8%) of clonal hematopoiesis in a cohort of 95 β-thalassemic patients [160]. It was suggested that life-long stress in the hematopoiesis could represent a pathogenetic mechanism favoring CH development in β-thalassemic patients [160].

## 7. Cost-Effectiveness of HSCT for Hemoglobinopathies

HSCT for hemoglobinopathies is considered a cost-effective and curative treatment, with an acceptable safety profile compared to lifelong standard care (including transfusions, iron chelation, hospitalizations, pharmaceuticals, medical support for acute events and medical treatments for organ damage), despite its high upfront cost.

HSCT offers a one-time cure, particularly for younger SCD and β-thalassemic patients who have a matched-related donor. HSCT improves the quality of life of both SCD and β-thalassemic patients, preventing pain crises, organ damage dependent on pathologic events related to SCD and β-thalassemia, and premature death. Furthermore, HSCT improves the social participation of SCD and β-thalassemic patients to an active social life.

The evaluation of the cost of allo-HSCT for SCD and β-thalassemic patients is challenging because charges depend on many variables, including type of transplant (matched-related donor HSCTs are generally less costly and more effective than matched-unrelated donor or haploidentical donor or PBSC or CB transplants), state regulations, regional health costs and insurance contracts [161]. The cost of an HSCT for SCD and β-thalassemia varies dramatically by location, ranging from $10,000 to 50,000 in some developing countries to $80,000 to 400,000 in the USA/Western Europe. A detailed analysis performed in the USA showed that the total cost of allo-HSCT in SCD patients ranges from $340,000 to $799,000, with a median total cost of $467,000 [162]. Several variables affect the cost of allo-HSCT in these patients: healthcare utilization was lower among recipients of matched sibling donor grafts and in patients early transplanted (young children), and this was due to a reduced post-transplant healthcare utilization [162]. A recent evaluation showed in the USA a total median cost of $130,000 for allo-HSCT in SCD patients encompassing a lapse of time from 6 months pre-transplant to two years post-transplant [163]. A comparative cost analysis carried out in the USA between HSCT vs. supportive care showed that the former is approximately equivalent to the cost of ten SCD hospital admissions [164].

Recent studies have shown that gene therapy represents an alternative approach to allo-HSCT to obtain a curative effect in SCD and β-thalassemic patients. The current procedure used in gene therapy studies implies the ex vivo gene modification of autologous stem cells and the reinfusion of genetically modified cells back to the patient. Particularly, the treatment process implies the following: initial evaluation involving patient selection and preparation; stem cell collection, involving the collection of HSCs from patient’s blood after HSC mobilization with G-CSF and Plerixafor; genetic modification implying genetic manipulation of autologous HSCs using either gene addition (lentiviral vectors) or gene editing (CRISPR) to correct the genetic defect; conditioning involving the hospitalization of the patient and preparation to HSC transplantation through administration of a conditioning regimen to clear abnormal cells from the bone marrow; infusion back into the patient of genetically modified HSCs that engraft and begin to produce functional erythroid cells, leading to a long-term cure; and clinical support, monitoring and follow-up [165,166]. Three gene therapy products have been approved for the treatment of β-hemoglobinopathies: Lyfgenia (lovotibeglogene autotemcel) and Zynteglo (betibeglogene autotemcel), which use lentiviral vectors to modify the patient’s stem cells, and Casgevy (exagamglogene autotemcel), which utilizes CRISPR gene editing of the patient’s stem cells [165,166].

Betibeglogene and Lovotibeglogene are two gene therapy products based on a lentiviral vector and use a technique of β-globin gene addition based on the ex vivo transfer of a human β-globin gene into patient’s HSCs/HPCs. The development of retroviral vectors allowing an efficient expression of the transduced β-globin gene into HSCs during erythroid differentiation along the erythroid lineage was made possible by the identification within the β-globin gene cluster region of regulatory elements required for high expression of the β-globin gene in adult erythroid cells. Both Lovotibeglogene and Betibeglogene contain the LentiGlobin BB305 vector encoding the β^A-T87Q^ globin (a mutant β-globin with functional properties similar to WT-β^A^ globin) and contain the locus control region (LCR) and the β-globin gene promoter to induce high expression of the β^A-T87Q^ globin in erythroid cells [167].

Betibeglogene was evaluated in β-thalassemia patients. The safety and efficacy of Betibeglogene was initially evaluated in a phase I/II clinical study, HGB-204 and HGB-205; in these studies, autologous HSCs/HPCs were ex vivo modified with Betibeglogene and then reinfused to patients after myeloablative Busulfan conditioning [168]. In these initial studies, the majority of treated patients reached a condition of transfusion independence or a drastic decreased of transfusion requirements (patients with β°/β° genotype) [168]. Two phase III clinical studies have supported the clinical efficacy of Betibeglogene. In the HGB-207 study, 20/22 β-thalassemic patients reached a long-term condition of transfusion independence with a median β^A-T87Q^ level of 8.7 g (dL at 12 months post-treatment and an average Hb level of 11.7 g/dL during transfusion independence [169]. The HBG-212 study enrolled 18 β-thalassemic patients, and 89% of them reached and maintained a condition of transfusion independence [170]. Importantly, in these studies, no serious events directly related to gene therapy and deaths were observed [169,170]. Based on the successful outcome of these studies, Betibeglogene was approved for the treatment of adult and pediatric β-thalassemic patients who require regular RBC transfusion support.

Levotibeglogene was investigated in clinical studies involving SCD patients; the lentiviral vector present in Levotibeglogene is identical to that present in Betibeglogene: the β^A-T87Q^ globin encoded by this vector sterically inhibits HbS polymerization and RBC sickling. Levotibeglogene was initially explored in three SCD patients showing persistent in vivo expression of the transduced β^A-T87Q^ globin, with a level of HbA^T87Q^ of 50% of total hemoglobin and cessation of sickle crises; two patients achieved a condition of transfusion independence, and one patient displayed a decrease in transfusion need [171]. A phase III clinical trial explored 36 SCD patients treated with a single infusion of autologous CD34^+^ cells transduced with Levotibeglogene: total Hb levels increased from 8.5 g/dL to 11 g/dL or higher, β^A-T87Q^ mean levels were 5.2 g/dL, contributing at least 40% of total Hb [172]. All treated patients displayed resolution of severe vaso-occlusive events and reduction in hemolysis [172]. The favorable outcome of this study has led to the authorization of Levotibeglogene for the treatment of patients with severe SCD.

The Exagamglogene product is based on a gene editing therapy approach; gene editing is a recently developed technology suitable to modify genetic information in patients with hemoglobinopathies. This technology, at variance with gene addition studies, does not need the generation of a lentiviral vector and the introduction of exogenous DNA. Two different approaches of gene editing have been explored in patients with β-hemoglobinopathies: gene editing for correction of single-nucleotide mutations, such as the β-globin mutation of SCD, and gene editing of regulatory DNA sequences (such as the DNA sequence of a site regulating the expression of BCL11A, a repressor of γ-globin gene expression in adult erythroid cells) reactivating fetal hemoglobin (HbF) synthesis in erythroid cells of SCD and β-thalassemic patients [167]. Clinical studies based on the gene editing of a DNA element regulating the expression of BCL11A in adult erythroid cells showed a consistent reactivation of HbF synthesis in adult erythroid cells, leading to a significant therapeutic effect in both SCD and β-thalassemia patients. An initial pilot study provided evidence supporting both the safety and the efficacy of Exagamglogene in SCD and β-thalassemic patients [156]. The CLIMB-THAL 111 and CLIMB-SCD 122 studies provided initial evidence in one β°/β° thalassemic and β^S^/β^S^ SCD patients that infusion of gene-edited autologous CD34^+^ cells induced in vivo after transplantation the production of an erythroid progeny producing high HbF levels; both these patients became transfusion-independent [173]. The phase III clinical study CLIMB-THAL 111 evaluated the safety and the efficacy of Exagamglogene-edited autologous CD34^+^ cells in 52 β-thalassemic patients, showing that 91% of these patients become transfusion-independent after gene therapy, with a mean Hb level of 13.1 g/dL and a mean HbF level of 11.9 g/dL; 9% of the treated patients did not reach transfusion independence but markedly decreased their transfusion needs [174]. The phase III CLIMB-SCD 121 trial explored the safety and efficacy of Exagamglogene-edited autologous CD34^+^ cells in 44 transfusion-dependent SCD patients; the majority (38/44) of treated patients were free of hospitalizations for severe vaso-occlusive events; all patients became transfusion-independent, and their Hb content increased from <9 g/dL to 12.5 g/dL, with 44.5% of HbF [175]. These studies were also extended to pediatric patients (age ranging from 2 to 11 years) with transfusion-dependent SCD or β-thalassemia, with a consistent and comparable clinical benefit [176]. The safety profile observed in these studies was consistent with Busulfan conditioning and autologous HSCT.

The availability of two different curative approaches for both SCD and β-thalassemic patients raises the need for a comparison between allo-HSCT and gene therapy treatments at the level of costs, efficacy, safety and patients’ eligibility. Several considerations related to the cost (the cost of allo-HSCT is five- or six-fold lower than the cost of approved gene therapies for β-hemoglobinopathies) and to the long-term effects (that are known for allo-HSCT for both SCD and β-thalassemic patients, but are only in part established for gene therapy treatments) favor allo-HSCT over gene therapy treatments [147]. A group of experts in hemoglobinopathies and/or in HSCT and gene therapy of the EHA and EBMT has recently proposed criteria for an optimal selection of patients with hemoglobinopathies for gene therapy studies [177]. These experts have proposed to restrict access to gene therapy to SCD patients in good clinical conditions with no-HLA-matched donor available, without irreversible severe organ impairment, and able to tolerate a myeloablative conditioning regimen [177]. Medical centers providing gene therapy must evaluate each SCD or β-thalassemic patient through a process ensuring ethical, systematic allocation of each patient either to HSCT or to gene therapy.

The existence of two curative therapies for β-hemoglobinopathies now may offer the opportunity to some patients who have experienced a failure to cure SCD or β-thalassemia with HSCT of a second curative treatment based on gene therapy [144]. However, this opportunity must be considered with caution since exposure to inflammatory stress and conditioning chemotherapy may compromise the fitness of HSCs, reduce the hematopoietic reserve, accelerate HSC aging, and promote the accumulation of deleterious genetic alterations, all of which may negatively affect the efficacy and the safety of gene therapy [144].

## 8. Conclusions

HSCT is the only proven cure for SCD and β-thalassemia and is based on the replacement of pathological HSCs with healthy donor cells, offering excellent outcomes in young patients with matched sibling donors but carrying a significant risk of transplant-related infections and mortality, thus requiring a careful risk–benefit assessment for eligible patients. The studies carried out in the last three decades have clearly shown that three parameters are key determinants for success of allo-HSCT in patients with β-hemoglobinopathies: donor match (with best results offered by matched sibling donors); patient’s age (with younger children < 8 years old offering the best results) and health (patients without organ damage have significantly better outcomes); and early intervention (HSCT before development of severe complications improves survival).

Since only 15–20% of patients have a sibling-matched donor, to expand the opportunities of allo-HSCT for patients with β-hemoglobinopathies, transplants have been developed using matched-unrelated donors or other family members (haploidentical) as donors. These HSCTs carry higher risks related to the lower incomplete immunological compatibility; however, strategies have been developed in the last two decades to reduce the risk of graft failure and to prevent aGVHD and cGVHD, with a consequent improvement of the outcomes of allo-HSCT with unrelated matched donors or with haploidentical donors. Furthermore, the development of efficient strategies of reduced-intensity conditioning and of immunosuppression has improved the outcomes of allo-HSCT in adult patients with β-hemoglobinopathies.

Gene therapy based on gene addition or gene editing strategies has led to the development of additional curative therapies for SCD and β-thalassemia, thus raising the problem of the selection of SCD and β-thalassemia patients for allo-HSCT or for gene therapy. The adoption of gene therapy treatments of β-hemoglobinopathies should be limited to patients who do not have HLA-matched donors and is strongly limited by its high cost, five to six-fold higher than HSCT, and its applicability to only a few highly specialized medical centers.

Finally, it is important to emphasize that both allo-HSCT and ex vivo gene therapies require a specialized infrastructure and considerable expertise for an HSCT, which are available only in developed countries and not in the low- and middle-income countries where SCD and β-thalassemia are mostly prevalent.

## Data Availability

No new data were created or analyzed in this study. Data sharing is not applicable to this article.
